# Supplementary material for: Canopy functional trait variation across Earth’s tropical forests
Source: Nature. 2025 Mar 5;641(8061):129–36. doi: 10.1038/s41586-025-08663-2 (PMC12043511; doi:10.1038/s41586-025-08663-2)
Supplement: Supplementary file 2 — Reporting Summary [file 41586_2025_8663_MOESM2_ESM.pdf]

nature portfolio

Corresponding author(s): Jesús Aguirre Gutiérrez

Last updated by author(s): Dec 15, 2024

Reporting Summary

Nature Portfolio wishes to improve the reproducibility of the work that we publish. This form provides structure for consistency and transparency in reporting. For further information on Nature Portfolio policies, see our Editorial Policies and the Editorial Policy Checklist.

Statistics

For all statistical analyses, confirm that the following items are present in the figure legend, table legend, main text, or Methods section.

n/a	Confirmed	
☐	☒	The exact sample size (*n*) for each experimental group/condition, given as a discrete number and unit of measurement
☐	☒	A statement on whether measurements were taken from distinct samples or whether the same sample was measured repeatedly
☐	☒	The statistical test(s) used AND whether they are one- or two-sided<br>*Only common tests should be described solely by name; describe more complex techniques in the Methods section.*
☐	☒	A description of all covariates tested
☐	☒	A description of any assumptions or corrections, such as tests of normality and adjustment for multiple comparisons
☐	☒	A full description of the statistical parameters including central tendency (e.g. means) or other basic estimates (e.g. regression coefficient) AND variation (e.g. standard deviation) or associated estimates of uncertainty (e.g. confidence intervals)
☐	☒	For null hypothesis testing, the test statistic (e.g. *F*, *t*, *r*) with confidence intervals, effect sizes, degrees of freedom and *P* value noted<br>*Give P values as exact values whenever suitable.*
☐	☒	For Bayesian analysis, information on the choice of priors and Markov chain Monte Carlo settings
☒	☐	For hierarchical and complex designs, identification of the appropriate level for tests and full reporting of outcomes
☐	☒	Estimates of effect sizes (e.g. Cohen's *d*, Pearson's *r*), indicating how they were calculated

Our web collection on statistics for biologists contains articles on many of the points above.

Software and code

Policy information about availability of computer code

Data collection	No software was used to collect data
Data analysis	All analyses were carried in Google EarthEngine v. 2024 and the R statistical environment R version 4.0.5 (2021-03-31). RandomForest v. 4.7-1.1, Ranger V. 0.15.1, Princomp v. 4.3.1 , Funspace v.0.2.2

For manuscripts utilizing custom algorithms or software that are central to the research but not yet described in published literature, software must be made available to editors and reviewers. We strongly encourage code deposition in a community repository (e.g. GitHub). See the Nature Portfolio guidelines for submitting code & software for further information.

Data

Policy information about availability of data

All manuscripts must include a data availability statement. This statement should provide the following information, where applicable:

- Accession codes, unique identifiers, or web links for publicly available datasets
- A description of any restrictions on data availability
- For clinical datasets or third party data, please ensure that the statement adheres to our policy

To comply with the original data owners' requirements, the plant functional traits and vegetation census data that support the findings of this study are available from their sources, GEM 31 at gem.tropicalforests.ox.ac.uk, and ForestPlots 32, 61, 62, www.ForestPlots.net and Diaz et al. 33. Given data sovereignty from the original data owners raw data on vegetation censuses and trait data are not publicly available but can be requested by contacting all researchers through the

ForestPlots 32, 61, 62 data request protocol described in forestplots.net/en/join-forestplots/working-with-data. The processed maps with community level trait predictions from this study are available as an app in Google Earth Engine at https://pantropicalanalysis.users.earthengine.app/view/pantropical-traits-aguirre-gutierrez-2025. Other environmental and plant data are available from their original sources: BIEN (bien.nceas.ucsb.edu), SoilGrids (https://soilgrids.org) and RESOLVE Ecoregions (https://ecoregions.appspot.com), Satellite data from the Senitnel-2 is freely available from the Google EarthEngine platform (https://developers.google.com/earth-engine/datasets/catalog/COPERNICUS_S2_SR_HARMONIZED).
R code for graphics and analyses is available on Zenodo DOI 10.5281/zenodo.14509493 (ref. 86).

Human research participants

Policy information about studies involving human research participants and Sex and Gender in Research.

Reporting on sex and gender	N/A
Population characteristics	N/A
Recruitment	N/A
Ethics oversight	N/A

Note that full information on the approval of the study protocol must also be provided in the manuscript.

Field-specific reporting

Please select the one below that is the best fit for your research. If you are not sure, read the appropriate sections before making your selection.

☐ Life sciences ☐ Behavioural & social sciences ☒ Ecological, evolutionary & environmental sciences

For a reference copy of the document with all sections, see nature.com/documents/nr-reporting-summary-flat.pdf

Ecological, evolutionary & environmental sciences study design

All studies must disclose on these points even when the disclosure is negative.

Study description	In this manuscript, we apply novel approaches encompassing field ecology and satellite remote sensing to produce and interpret for the first time spatially explicit predictions of 13 plant canopy functional traits covering the tropical forests distributed across the Americas, Africa, South East Asia and Australia. To tackle our questions, we field collected a comprehensive and unique pantropical dataset of highly relevant morphological, structural and leaf chemistry plant functional traits and linked them to >1800 unique plots with vegetation census which are recorded in across tropical forests.
Research sample	>1800 permanent plots
Sampling strategy	The sampling strategy was to collect plant functional traits for species that occupied at least 70% of the basal area of the vegetation plot and gather census data for as many undisturbed vegetation plots as possible.
Data collection	Local collaborators in each country and other co-authors from this manuscript collected the vegetation and census data. All vegetation census data is saved in the ForestPlots.net database (Leeds University) and the functional traits in the GEM traits database from the University of Oxford.
Timing and spatial scale	Most of the vegetation plots are of 1ha and their census time varies per region as these have been collected at different points in time but all censused in the last 5-10 years. The plant functional traits have been collected in most of the censused vegetation plots and covering at least 70% of the species basal area. The functional traits have been collected in tropical forests across the the four tropical continents.
Data exclusions	No data were excluded.
Reproducibility	Attempts to reproduce the analysis were successful.
Randomization	This is not relevant as we collected as much information as possible to make our analysis robust and did not need to randomize our samples for our analysis.
Blinding	Blinding was not relevant for our analysis.

Did the study involve field work? ☐ Yes ☒ No

Reporting for specific materials, systems and methods

We require information from authors about some types of materials, experimental systems and methods used in many studies. Here, indicate whether each material, system or method listed is relevant to your study. If you are not sure if a list item applies to your research, read the appropriate section before selecting a response.

Materials & experimental systems

n/a	Involved in the study
☒	☐ Antibodies
☒	☐ Eukaryotic cell lines
☒	☐ Palaeontology and archaeology
☒	☐ Animals and other organisms
☒	☐ Clinical data
☒	☐ Dual use research of concern

Methods

n/a	Involved in the study
☒	☐ ChIP-seq
☒	☐ Flow cytometry
☒	☐ MRI-based neuroimaging
