# Supplementary material for: Canopy functional trait variation across Earth’s tropical forests
Source: Nature. 2025 Mar 5;641(8061):129–36. doi: 10.1038/s41586-025-08663-2 (PMC12043511; doi:10.1038/s41586-025-08663-2)
Supplement: Supplementary file 1 — This file includes Supplementary Figs. 1–13, which provide the predicted distribution of community weighted mean (CWM) traits, and Supplementary Tables 1–7, which include details on traits used, description of vegetation plots used and model statistical results. [file 41586_2025_8663_MOESM1_ESM.pdf]

nature portfolio

https://doi.org/10.1038/s41586-025-08663-2

Supplementary information

Canopy functional trait variation across Earth's tropical forests

In the format provided by the authors and unedited

Supplemental Information

Canopy functional trait variation across Earth's tropical forests

Jesús Aguirre Gutiérrez [1, 2, *], Sami W. Rifai [3], Xiongjie Deng [1], Hans ter Steege[98, 99], Eleanor Thomson [1], Aretha Franklin Guimaraes [4], Sandra Muller [5], Joice Klipel [5], Sophie Fauset [6], Angelica F. Resende [7, 8], Göran Wallin [9, 1], Carlos A. Joly [10], Katharine Abernethy [12, 11], Stephen Adu-Bredu [13], Celice Alexandre Silva [14], Edmar Almeida de Oliveira [15], Danilo R. A. Almeida [7], Esteban Alvarez-Davila [16], Gregory P. Asner [17], Timothy R. Baker [18], Maíra Benchimol [19], Lisa Patrick Bentley [20], Erika Berenguer [1], Lilian Blanc [21, 22], Damien Bonal [23], Kauane Bordin [5], Robson Borges de Lima [24], Sabine Both [25], Jaime Cabezas Duarte [26, 27], Domingos Cardoso [28, 29], Haroldo C. de Lima [29], Larissa Cavalheiro [30], Lucas A. Cernusak [31], Nayane Cristina C. dos Santos Prestes [15], Antonio Carlos da Silva Zanzini [32], Ricardo José da Silva [14], Robson dos Santos Alves da Silva [14], Mariana de Andrade Iguatemy [29, 33], Tony César De Sousa Oliveira [34, 35], Benjamin Dechant [95, 96], Géraldine Derroire [21, 22, 36], Kyle G. Dexter [38, 37, 105], Domingos J. Rodrigues [30], Mário Espírito-Santo [39], Letícia Fernandes Silva [41, 40], Tomas Ferreira Domingues [34, 42], Joice Ferreira [43], Marcelo Fragomeni Simon [44], Cécile A. J. Girardin [1], Bruno Hérault [21, 22], Kathryn J. Jeffery [11], Sreejith Kalpuzha Ashtamoorthy [45], Arunkumar Kavidapadinjattathil Sivadasan [45], Bente Klitgaard [46], William F. Laurance [31], Maurício Lima Dan [47], William E. Magnusson [4], Eduardo Malta Campos-Filho [48], Rubens Manoel dos Santos [49], Angelo Gilberto Manzatto [50], Marcos Silveira [51], Ben Hur Marimon-Junior [52], Roberta E. Martin [17], Daniel Luis Mascia Vieira [44], Thiago Metzker [53, 54], William Milliken [55], Peter Moonlight [56], Marina Maria Moraes de Seixas [57], Paulo S. Morandi [58], Robert Muscarella [59], María Guadalupe Nava-Miranda [61, 60], Brigitte Nyirambangutse [62, 63], Jhonathan Oliveira Silva [64], Imma Oliveras Menor [1, 65], Pablo José Francisco Pena Rodrigues [29], Cinthia Pereira de Oliveira [24], Lucas Pereira Zanzini [67], Carlos A. Peres [68], Vignesh Punjayil [45], Carlos A. Quesada [69], Maxime Réjou-Méchain [70], Terhi Riutta [1, 71], Gonzalo Rivas-Torres [72], Clarissa Rosa [4], Norma Salinas [73], Rodrigo Scarton Bergamin [75, 74], Beatriz Schwantes Marimon [52], Alexander Shenkin [76], Priscyla Silva Rodrigues [64], Axa Emanuelle Simões Figueiredo [77], Queila Souza Garcia [78], Tereza Spósito [53], Danielle Storck-Tonon [79], Martin Sullivan [80], Martin Svátek [81], Wagner Tadeu Vieira Santiago [82], Yit Arn Teh [83], Prasad Theruvilparambil Sivan [45], Marcelo Trindade Nascimento [84], Elmar Veenendaal [85], Irire Casimir Zo-Bi [100], Marie Ruth Dago [100], Soulemane Traoré [100, 103], Marco Patacca [101], Vincyane Badouard [70, 100, 102], Samuel de Padua Chaves e Carvalho [104], Lee J. T. White [12, 11], Huanyuan Zhang-Zheng [1, 2], Etienne Zibera [87, 9], Joeri Alexander Zwerts [88], David F.R.P. Burslem [89], Miles Silman [91, 90], Jérôme Chave[97], Brian J. Enquist [92], Jos Barlow [93], Oliver L. Phillips [18], David A. Coomes [94], Yadvinder Malhi [1, 2]

[*] *Corresponding author*

[1] Environmental Change Institute, School of Geography and the Environment, University of Oxford, UK

[2] Leverhulme Centre for Nature Recovery, University of Oxford, UK

[3] School of Biological Sciences, University of Adelaide 5005, Adelaide, South Australia, Australia

[4] Coordenação de Biodiversidade, Instituto Nacional de Pesquisas da Amazônia, Av. Andre Araujo 2936, 69067-375 Manaus, AM, Brazil

[5] Plant Ecology Lab, Ecology Department, Universidade Federal do Rio Grande do Sul, Porto Alegre, Brazil

[6] School of Geography, Earth and Environmental Sciences, University of Plymouth, PL4 8AA, United Kingdom

[7] Department of Forest Sciences, "Luiz de Queiroz" College of Agriculture, University of São Paulo (USP/ESALQ), Piracicaba, SP, Brazil

[8] Biological and Environmental Sciences, University of Stirling, UK

[9] Department of Biological and Environmental Sciences, University of Gothenburg, P.O. Box 463, SE-405 30 Gothenburg, Sweden

[10] Universidade Estadual de Campinas, Instituto de Biologia, Departamento de Biologia Vegetal, Campinas, São Paulo, Brazil and Brazilian Platform on Biodiversity and Ecosystem Services/BPBES
[11] Biological and Environmental Sciences, University of Stirling, Stirling, UK.
[12] Institut de Recherche en Écologie Tropicale, Libreville, Gabon.
[13] CSIR-Forestry Research Institute of Ghana, University P.O. Box 63, Kumasi, Ghana
[14] Universidade do Estado de Mato Grosso, Brazil
[15] PPG Ecology and Conservation, Universidade do Estado de Mato Grosso, Nova Xavantina, Brazil
[16] Escuela de Ciencias Agrícolas, Pecuarias y Ambientales - ECAMPA, Universidad Nacional Abierta y a Distancia - Colombia
[17] Center for Global Discovery and Conservation Science, Arizona State University, Tempe, AZ, United States
[18] School of Geography, University of Leeds, United Kingdom
[19] Laboratório de Ecologia Aplicada à Conservacão, Departamento de Ciências Biológicas, Universidade Estadual de Santa Cruz, Ilhéus, Bahia 45662-900, Brazil
[20] Department of Biology, Sonoma State University, 1801 East Cotati Avenue, Rohnert Park, CA 94928, USA
[21] CIRAD, UPR Forêts et Sociétés, F-34398 Montpellier, France
[22] Forêts et Sociétés, Univ Montpellier, CIRAD, Montpellier, France
[23] INRAE, Université de Lorraine, AgroParisTech, UMR Silva, 54000 Nancy, France
[24] Laboratório de Manejo Florestal, Universidade do Estado do Amapá, Macapá, Brazil
[25] Environmental and Rural Science, University of New England, Armidale, 2351, NSW, Australia
[26] Jardín Botánico de Bogotá
[27] Universidad del Tolima
[28] Instituto de Biologia, Universidade Federal da Bahia, Salvador, Brazil
[29] Instituto de Pesquisas Jardim Botânico do Rio de Janeiro, Rua Pacheco Leão, 915, CEP 22460-030, Rio de Janeiro, Brazil
[30] Universidade Federal de Mato Grosso. Av. Alexandre Ferronato, 1200 - Res. Cidade Jardim, CEP: 78.550-728, Sinop, MT, Brazil
[31] College of Science and Engineering, James Cook University, Cairns, Queensland 4878, Australia
[32] Departamento de Engenharia Florestal, Universidade Federal de Lavras, Lavras, MG, Brazil
[33] Instituto Internacional para Sustentabilidade, Rio de Janeiro, Brazil
[34] Faculdade de Filosofia, Ciências e Letras de Ribeirão Preto
[35] Plant Ecology and Nature Conservation Group, Wageningen University
[36] Cirad, UMR EcoFoG (AgroParistech, CNRS, INRAE, Université des Antilles, Université de la Guyane), Campus Agronomique, Kourou, French Guiana
[37] Royal Botanic Garden Edinburgh, Edinburgh EH3 5LR, United Kingdom
[38] School of GeoSciences, University of Edinburgh, Edinburgh EH9 3FF, United Kingdom
[39] Departamento de Biologia Geral, Universidade Estadual de Montes Claros, Montes Claros-MG, Brazil
[40] Universidade Federal do Acre, Rio Branco, Acre, Brazil
[41] Universidade Paulista, Polo Rio Branco, Acre, Brazil
[42] Universidade de São Paulo, Brazil
[43] MCT/Museu Paraense Emílio Goeldi, Av. Magalhães Barata 376, São Braz, 66040-170 Belém, PA, Brazil
[44] Embrapa Recursos Genéticos e Biotecnologia, Brasília, Brazil
[45] Forest Ecology Department, KSCSTE-Kerala Forest Research Institute, Peechi, Thrissur, Kerala, India Pin: 680653
[46] Department for Accelerated Taxonomy, Royal Botanic Gardens, Kew, Surrey TW9 3AE, United Kingdom
[47] Centro de Pesquisa, Desenvolvimento e Inovação Sul, Instituto Capixaba de Pesquisa, Assistência Técnica e Extensão Rural, Rodovia João Domingos Zago, Km 2.5, Cachoeiro de Itapemirim 29323-000, Espírito Santo, Brazil
[48] Instituto Socioambiental
[49] Laboratório de Fitogeografia e Ecologia Evolutiva, Departamento de Ciências Florestais, Universidade Federal de Lavras

[50] Departamento de Biologia, Universidade Federal de Rondônia, Unir, Porto Velho, Brazil
[51] Centro de Ciências Biológicas e da Natureza, Universidade Federal do Acre, Br 364 km 4, Distrito Industrial, 69920-900, Rio Branco, Acre, Brazil
[52] Laboratório de Ecologia Vegetal (LABEV), Universidade do Estado de Mato Grosso, Nova Xavantina, Brazil
[53] IBAM - Instituto Bem Ambiental
[54] Myr Projetos Sustentáveis
[55] Royal Botanic Gardens, Kew, Surrey, TW9 3AE, United Kingdom
[56] Botany, School of Natural Sciences, Trinity College Dublin, Ireland
[57] Embrapa Amazônia Oriental, Trav. Dr. Enéas Pinheiro, s/n, CP 48, 66095-100 Belém, PA, Brazil
[58] Programa de Pós-graduação em Ecologia e Conservação, Universidade do Estado de Mato Grosso, Nova Xavantina, Brazil
[59] Plant Ecology and Evolution, Department of Ecology and Genetics, Uppsala University, Sweden
[60] Colegio de Ciencias y Humanidades, Universidad Juárez del Estado de Durango, México.
[61] Escuela Politécnica Superior de Ingeniería. Campus Terra. Universidad de Santiago de Compostela, España.
[62] Global Green Growth Institute, Rwanda Program, Kigali Heights, West Wing, 3rd Floor, Kigali, Rwanda
[63] University of Rwanda, KK 737 Street, Gikondo, Kigali, P.O. Box 4285, Kigali, Rwanda
[64] Colegiado de Ecologia, Universidade Federal do Vale do São Francisco-UNIVASF, Senhor do Bonfim, Brazil
[65] AMAP (Botanique et Modélisation de l'Architecture des Plantes et des Végétations), CIRAD, CNRS, INRA, IRD, Université de Montpellier, Montpellier, France
[66] Laboratório de Manejo Florestal, Universidade do Estado do Amapá, Macapá, Brazil
[67] Departamento de Engenharia Florestal, Universidade do Estado de Mato Grosso, Caceres, MT, Brazil
[68] School of Environmental Sciences, University of East Anglia, Norwich, UK
[69] Coordenação de Dinâmica Ambiental, Instituto Nacional de Pesquisas da Amazônia, Manaus, Brazil
[70] AMAP, Univ. Montpellier, IRD, CNRS, CIRAD, INRAE, Montpellier, France
[71] Department of Geography, University of Exeter, Exeter, EX4 4RJ, UK
[72] Estación de Biodiversidad Tiputini, Colegio de Ciencias Biológicas y Ambientales, Universidad San Francisco de Quito, Quito, Ecuador
[73] Institute for Nature Earth and Energy, Pontificia Universidad Católica del Perú, Avenida Universitaria 1801, San Miguel, Lima, Perú
[74] Birmingham Institute of Forest Research (BIFoR), University of Birmingham, United Kingdom
[75] School of Geography, Earth and Environmental Sciences, University of Birmingham, United Kingdom
[76] School of Informatics, Computing, and Cyber Systems, Northern Arizona University, Flagstaff, AZ, USA
[77] Departamento de Engenharia Florestal, Universidade de Brasília, Brasília 70910-000, DF, Brazil
[78] Instituto de Ciências Biológicas, Departamento de Botânica, Universidade Federal de Minas Gerais, Brazil
[79] Programa de Pós-graduação em Ambiente e Sistemas de Produção Agrícola. Universidade do Estado de Mato Grosso, Brazil
[80] Department of Natural Sciences, Manchester Metropolitan University, Manchester, UK
[81] Department of Forest Botany, Dendrology and Geobiocoenology, Faculty of Forestry and Wood Technology, Mendel University in Brno, Brno, Czech Republic
[82] CESAM - Centro de Estudos do Ambiente e do Mar, Departamento de Biologia. Pesquisador Colaborador, Universidade de Aveiro, Aveiro, Portugal
[83] School of Natural and Environmental Sciences, Newcastle University, Newcastle Upon Tyne, UK
[84] Laboratório de Ciências Ambientais, CBB, Universidade Estadual do Norte Fluminense Darcy Ribeiro, Campos dos Goytacazes, RJ, Brazil
[85] Plant Ecology & Nature Conservation Group, Wageningen University
[86] Institut de Recherche en Écologie Tropicale, Libreville, Gabon

[87] School of Forestry and Biodiversity and Biological Sciences, College of Agriculture, Animal Sciences and Veterinary Medicine, University of Rwanda, P.O. Box 210 Musanze, Rwanda
[88] Utrecht University
[89] School of Biological Sciences, University of Aberdeen, St Machar Drive, Aberdeen AB24 3UU, Scotland, U.K.
[90] Center for Energy, Environment, and Sustainability, Wake Forest University, Winston-Salem, NC 27109, USA
[91] Department of Biology, Wake Forest University, Winston-Salem, NC 27109, USA
[92] Department of Ecology and Evolutionary Biology, University of Arizona, Tucson, AZ, USA
[93] Lancaster Environment Centre, Lancaster University, Lancaster LA1 4YQ, UK
[94] Conservation Research Institute and Department of Plant Sciences, University of Cambridge, Cambridge CB2 3QZ, UK
[95] German Centre for Integrative Biodiversity Research (iDiv), Halle-Jena-Leipzig, Leipzig, Germany
[96] Leipzig University, Leipzig, Germany
[97] Laboratoire Evolution et Diversité Biologique, CNRS, UPS, IRD, Université Paul Sabatier, Toulouse, France
[98] Naturalis Biodiversity Center, PO Box 9517, Leiden, 2300 RA, The Netherlands
[99] Quantitative Biodiversity Dynamics, Utrecht University, Padualaan 8, Utrecht, 3584 CH, The Netherlands
[100] Institut National Polytechnique Félix Houphouët-Boigny, INP-HB, Yamoussoukro, Côte d'Ivoire
[101] Forest Ecology and Forest Management Group, Wageningen University and Research, Wageningen, the Netherlands
[102] Cirad, UMR EcoFoG (AgroParistech, CNRS, INRAE, Université des Antilles, Université de la Guyane), Campus Agronomique, Kourou, French Guiana
[103] Ministry of Water and Forests, Abidjan, Ivory Coast
[104] Federal Rural University of Rio de Janeiro, Seropedica, Brazil
[105]Department of Life Sciences and Systems Biology, University of Turin, Turin, Italy

This file contains the Supplementary Figs. 1–13 and Supplementary Tables 1–7.

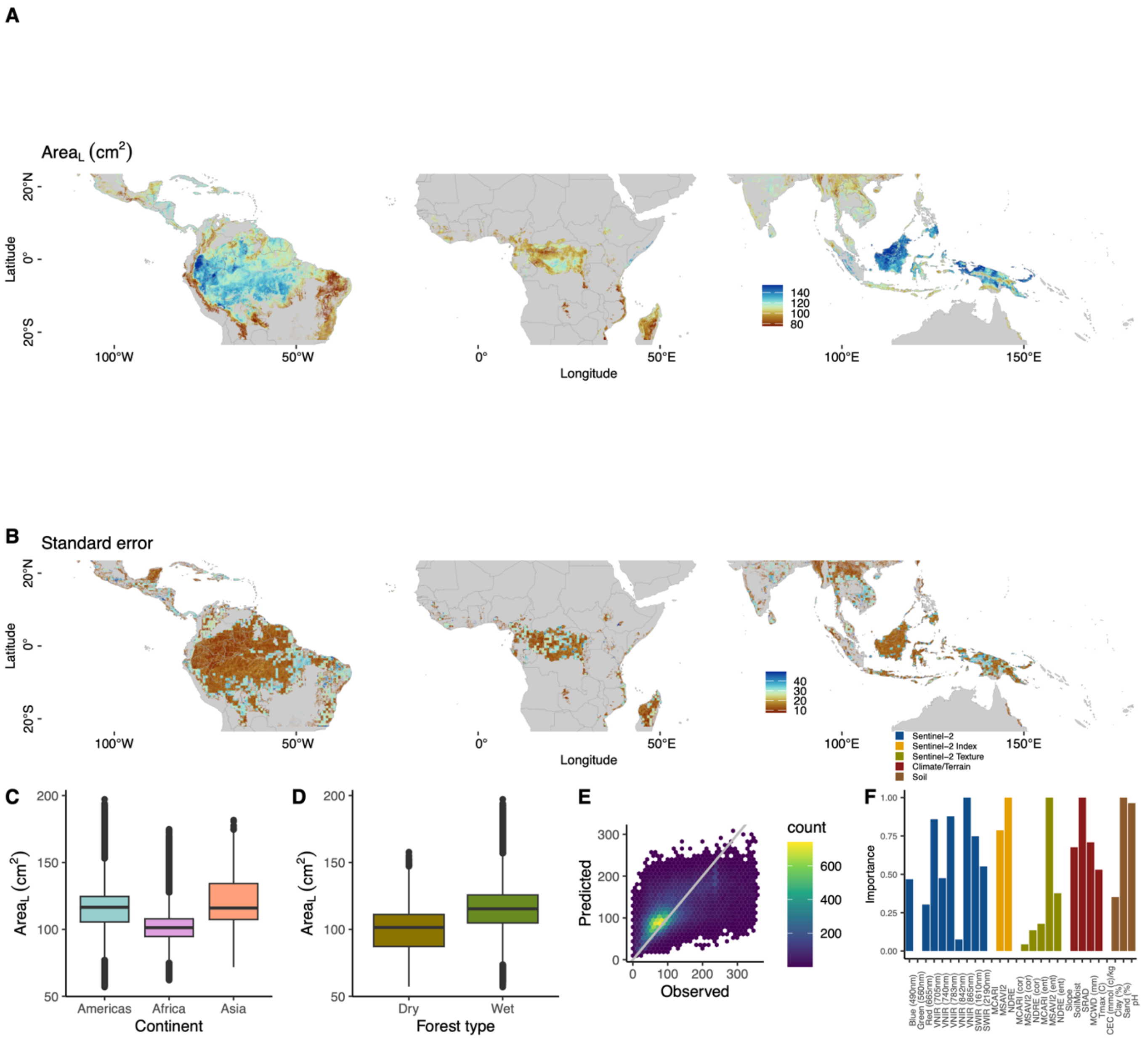

Supplementary Figure 1. Predicted distribution of community weighted mean (CWM) leaf area (Area$_L$). (A) Shows the predicted spatial distribution of this tree trait. (B) Standard Error of predicted distribution of CWM. (C) Shows boxplots for the CWM trait distribution values for tropical America, Africa and Asia extracted from the spatial predictions in (A). (D) Shows boxplots for the CWM trait distribution values for tropical dry (Dry) and tropical wet (Wet) forests across the tropical American, African and Asian forests classified and extracted from the spatial predictions in (A) using the RESOLVE Ecoregions dataset (ecoregions.appspot.com). (E) Shows the predicted versus observed CWM trait values with lighter colours depicting a higher count of pixels (i.e. area). (F) Shows the variable importance values, ranging from 0 (no importance) to 1 (high importance), of the covariates used in the Random Forest model for predicting CWM traits, with blue colour showing importance for Senitnel-2 spectral bands, yellow for vegetation indices, green for texture metrics, maroon for climate and terrain variables and brown for soil related variables. In (A) and (B) red to orange show areas with low to intermediate trait CWM or prediction standard error respectively, while light to dark blue depicts areas with intermediate to high CWM trait values or standard error trait predictions respectively. For visualisation purposes we excluded the extreme lowest and highest 1% of values in the maps. In (C) and (D) the horizontal black line depicts the median CWM trait value and vertical lines show the whiskers extending to the largest CWM trait value or not further than 1.5 times the interquartile range and filled circles above and below the vertical line depict outlier values. For statistical model results of (A) and (B) see Supplementary Table 3. For the significance of differences between CWM trait mean values in (C) and (D), obtained using a T-test, see Supplementary Tables 4 and 5.

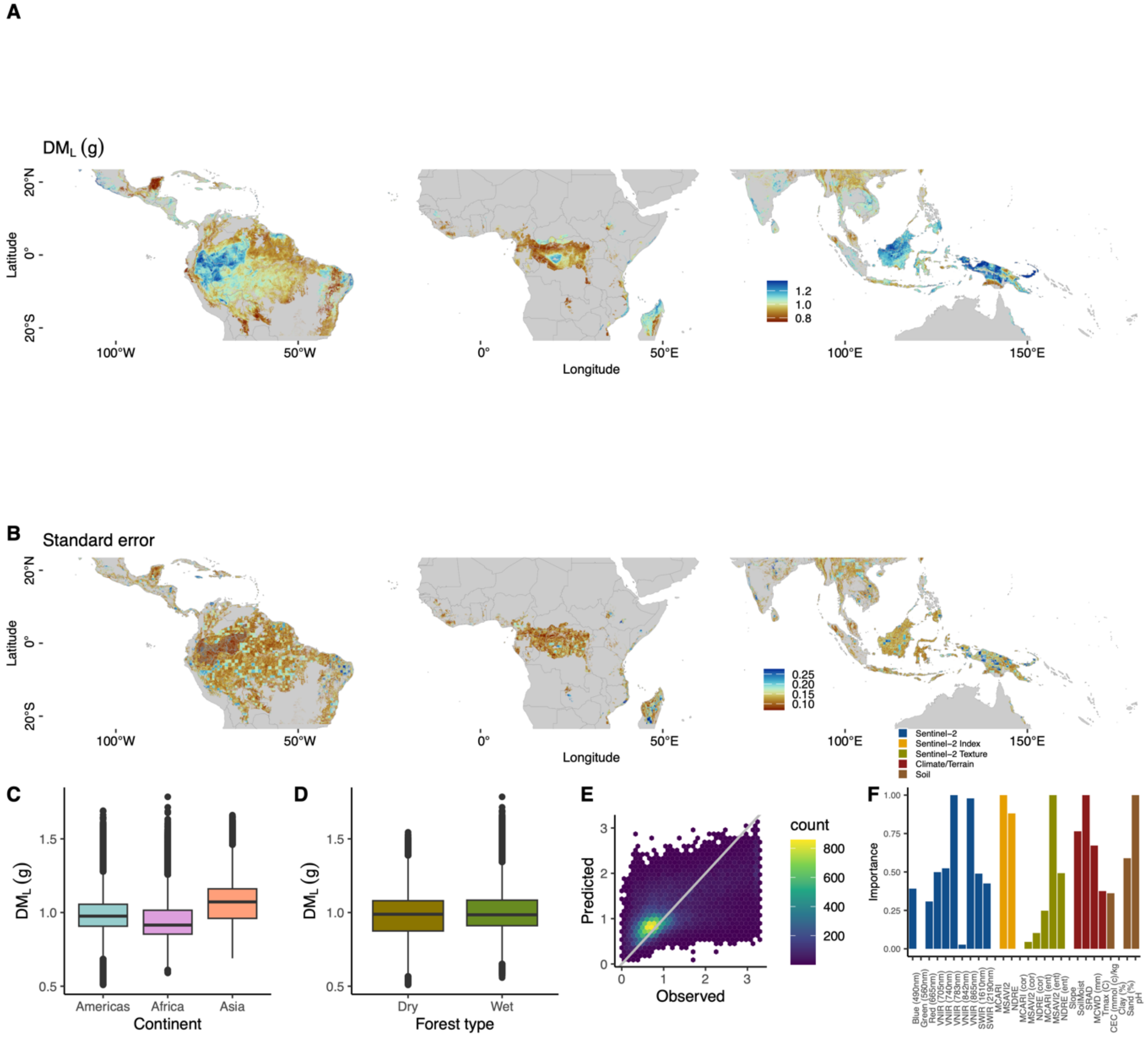

Supplementary Figure 2. Predicted distribution of community weighted mean (CWM) leaf dry mass (DM*L*). (A) Shows the predicted spatial distribution of this tree trait. (B) Shows boxplots for the CWM trait distribution values for tropical American (Am), African (Af) and Asian (As) forests extracted from the spatial predictions in (A). (C) Shows boxplots for the CWM trait distribution values for tropical dry (Dry) and tropical wet (Wet) forests across the tropical American, African and Asian forests classified and extracted from the spatial predictions in (A) using the RESOLVE Ecoregions dataset (ecoregions.appspot.com). (D) Shows the predicted versus observed CWM trait values with lighter colours depicting a higher count of pixels or area. (E) Shows the variable importance values, ranging from 0 (no importance) to 1 (high importance), of the covariates used in the Random Forest model for predicting CWM traits, with blue colour showing importance for Senitnel-2 spectral bands, yellow for vegetation indices, green for texture metrics, maroon for climate and terrain variables and brown for soil related variables. In (A) red to orange show areas with low to intermediate trait values, while light to dark blue depict areas with intermediate to high trait values. In (B) and (C) the horizontal black line depicts the median CWM trait value and vertical lines show the whiskers extending to the largest CWM trait value or not further than 1.5 times the interquartile range and filled circles above and below the vertical line depict outlier values. The map of prediction uncertainty (standard error) is shown in Supplementary Figure 15. For the significance of differences between CWM trait mean values in (B) and (C), obtained using a T-test, see Supplementary Tables 4 and 5.

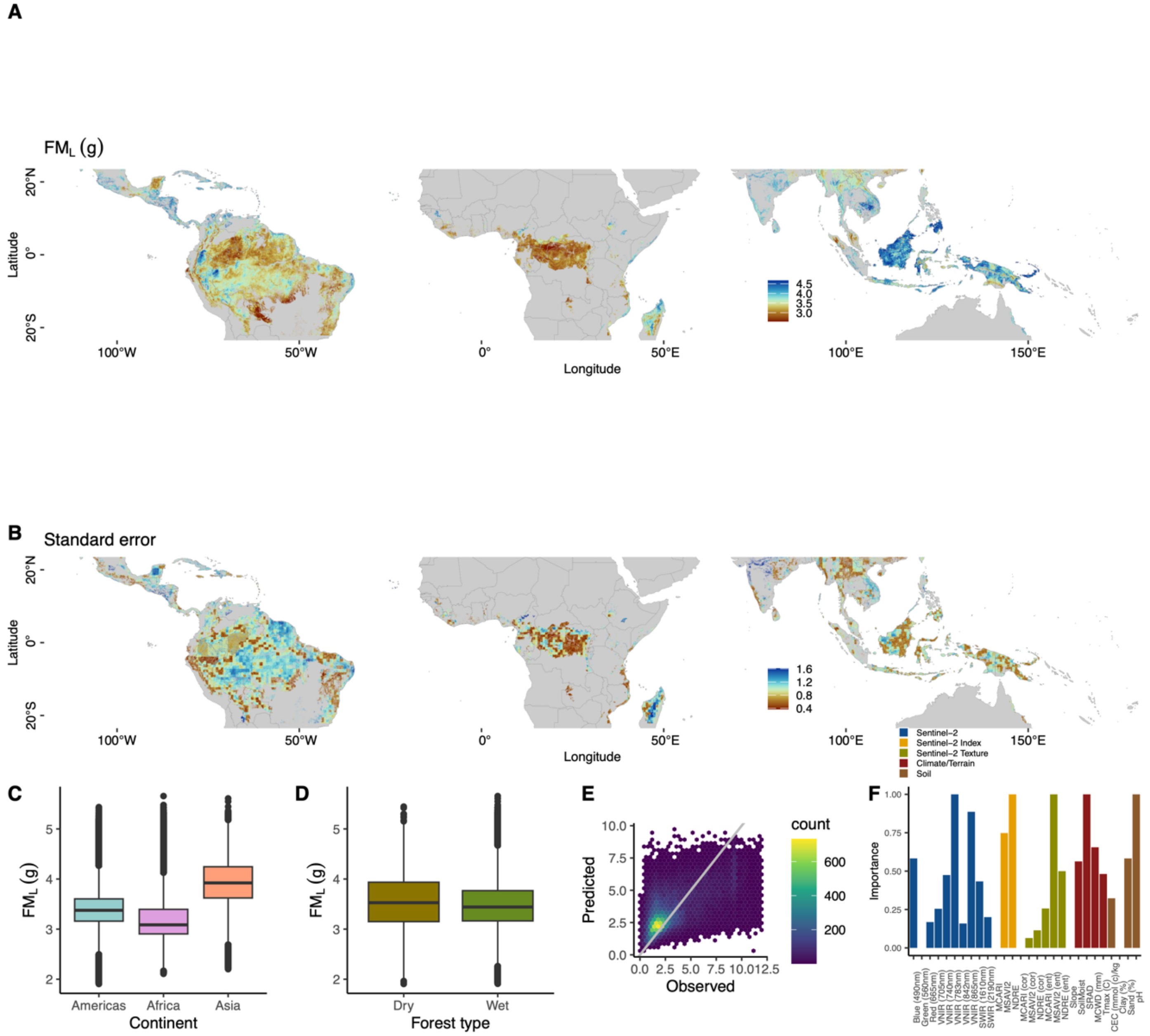

Supplementary Figure 3. Predicted distribution of community weighted mean (CWM) leaf fresh mass (FM*L*). (A) Shows the predicted spatial distribution of this tree trait. (B) Standard Error of predicted distribution of CWM. (C) Shows boxplots for the CWM trait distribution values for tropical America, Africa and Asia extracted from the spatial predictions in (A). (D) Shows boxplots for the CWM trait distribution values for tropical dry (Dry) and tropical wet (Wet) forests across the tropical American, African and Asian forests classified and extracted from the spatial predictions in (A) using the RESOLVE Ecoregions dataset (ecoregions.appspot.com). (E) Shows the predicted versus observed CWM trait values with lighter colours depicting a higher count of pixels (i.e. area). (F) Shows the variable importance values, ranging from 0 (no importance) to 1 (high importance), of the covariates used in the Random Forest model for predicting CWM traits, with blue colour showing importance for Senitnel-2 spectral bands, yellow for vegetation indices, green for texture metrics, maroon for climate and terrain variables and brown for soil related variables. In (A) and (B) red to orange show areas with low to intermediate trait CWM or prediction standard error respectively, while light to dark blue depicts areas with intermediate to high CWM trait values or standard error trait predictions respectively. For visualisation purposes we excluded the extreme lowest and highest 1% of values in the maps. In (C) and (D) the horizontal black line depicts the median CWM trait value and vertical lines show the whiskers extending to the largest CWM trait value or not further than 1.5 times the interquartile range and filled circles above and below the vertical line depict outlier values. For statistical model results of (A) and (B) see Supplementary Table 3. For the significance of differences between CWM trait mean values in (C) and (D), obtained using a T-test, see Supplementary Tables 4 and 5.

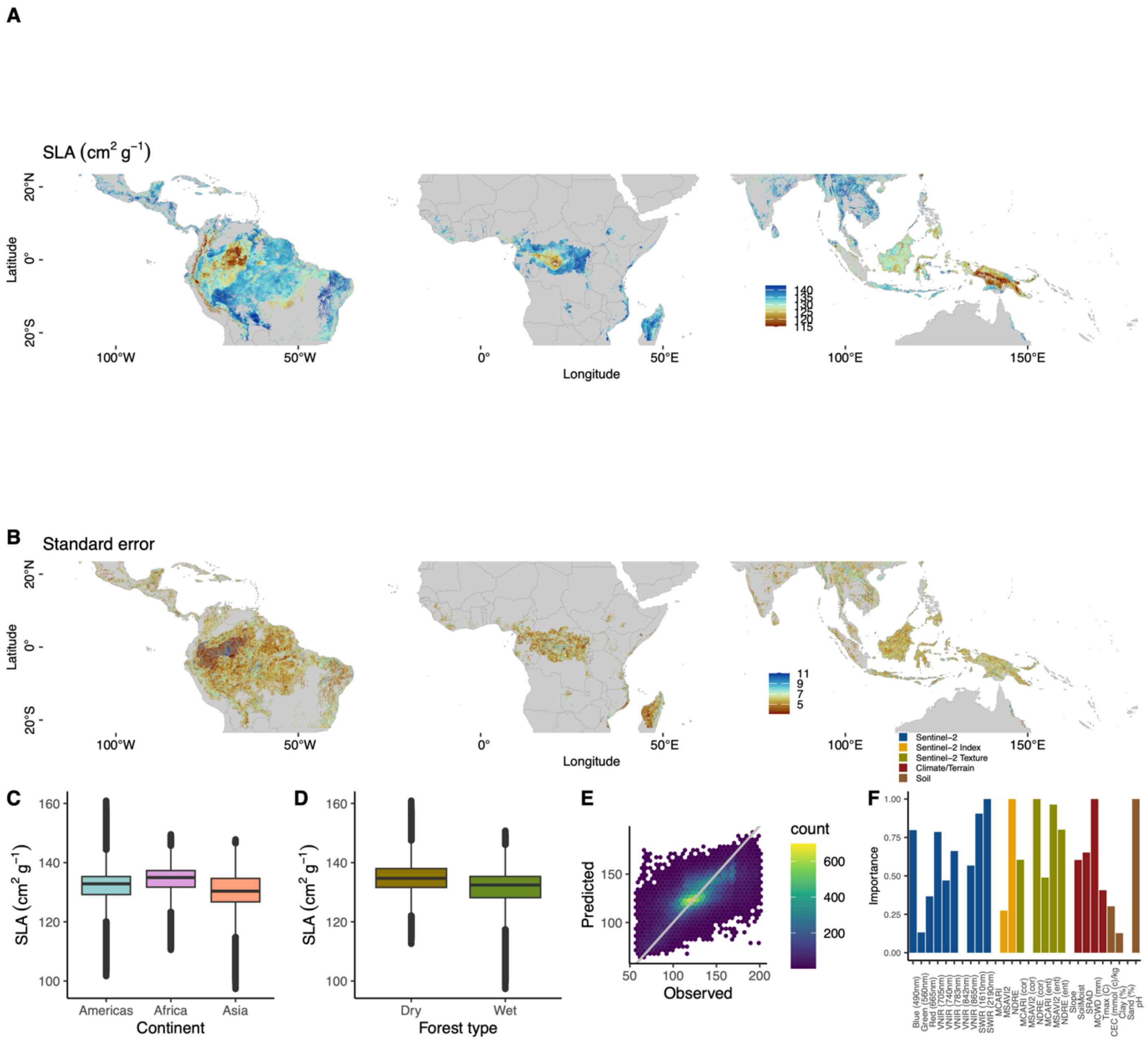

Supplementary Figure 4. Predicted distribution of community weighted mean (CWM) specific leaf area (SLA). (A) Shows the predicted spatial distribution of this tree trait. (B) Standard Error of predicted distribution of CWM. (C) Shows boxplots for the CWM trait distribution values for tropical America, Africa and Asia extracted from the spatial predictions in (A). (D) Shows boxplots for the CWM trait distribution values for tropical dry (Dry) and tropical wet (Wet) forests across the tropical American, African and Asian forests classified and extracted from the spatial predictions in (A) using the RESOLVE Ecoregions dataset (ecoregions.appspot.com). (E) Shows the predicted versus observed CWM trait values with lighter colours depicting a higher count of pixels (i.e. area). (F) Shows the variable importance values, ranging from 0 (no importance) to 1 (high importance), of the covariates used in the Random Forest model for predicting CWM traits, with blue colour showing importance for Senitnel-2 spectral bands, yellow for vegetation indices, green for texture metrics, maroon for climate and terrain variables and brown for soil related variables. In (A) and (B) red to orange show areas with low to intermediate trait CWM or prediction standard error respectively, while light to dark blue depicts areas with intermediate to high CWM trait values or standard error trait predictions respectively. For visualisation purposes we excluded the extreme lowest and highest 1% of values in the maps. In (C) and (D) the horizontal black line depicts the median CWM trait value and vertical lines show the whiskers extending to the largest CWM trait value or not further than 1.5 times the interquartile range and filled circles above and below the vertical line depict outlier values. For statistical model results of (A) and (B) see Supplementary Table 3. For the significance of differences between CWM trait mean values in (C) and (D), obtained using a T-test, see Supplementary Tables 4 and 5.

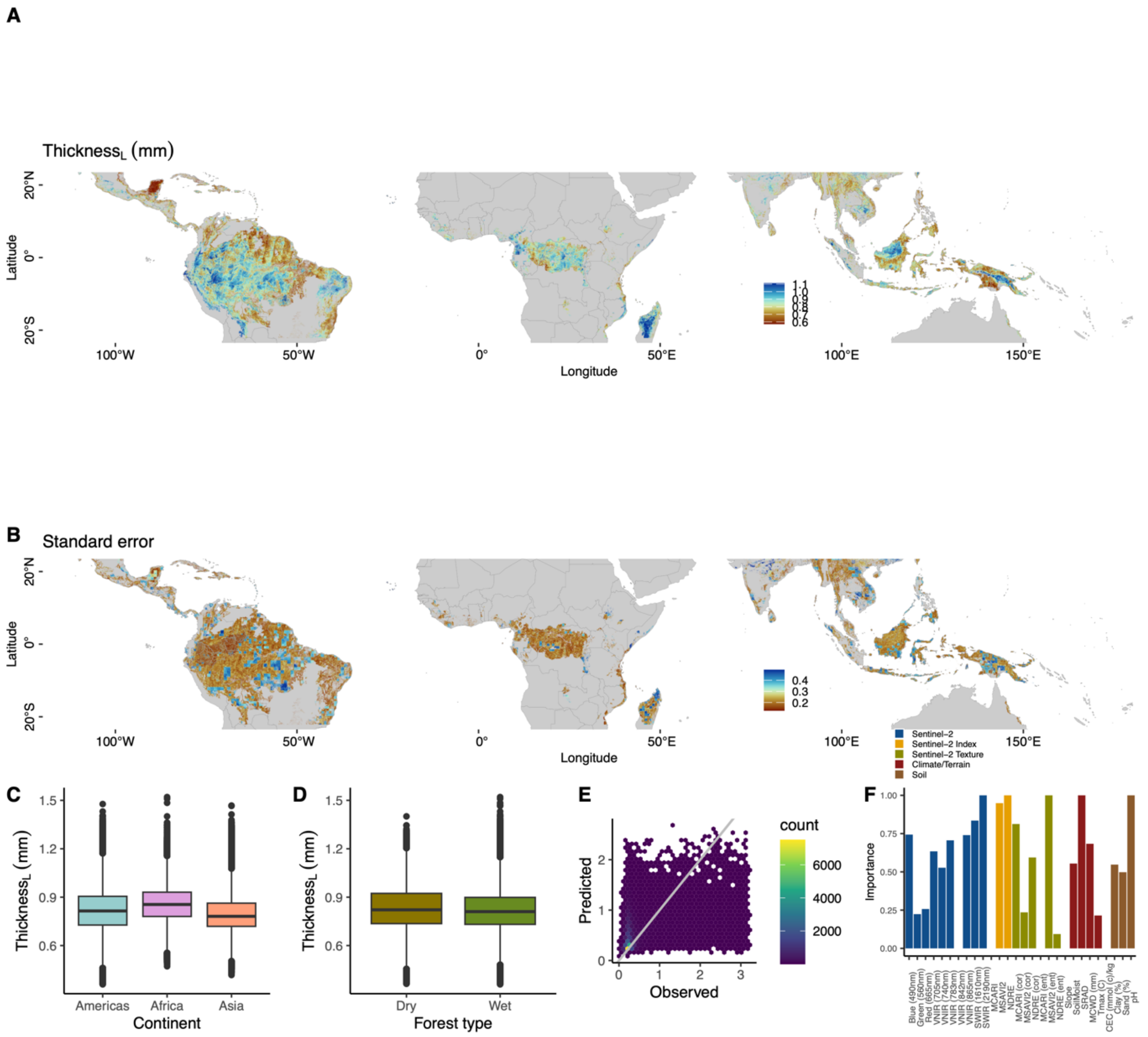

Supplementary Figure 5. Predicted distribution of community weighted mean (CWM) leaf thickness (Thickness$_L$). (A) Shows the predicted spatial distribution of this tree trait. (B) Standard Error of predicted distribution of CWM. (C) Shows boxplots for the CWM trait distribution values for tropical America, Africa and Asia extracted from the spatial predictions in (A). (D) Shows boxplots for the CWM trait distribution values for tropical dry (Dry) and tropical wet (Wet) forests across the tropical American, African and Asian forests classified and extracted from the spatial predictions in (A) using the RESOLVE Ecoregions dataset (ecoregions.appspot.com). (E) Shows the predicted versus observed CWM trait values with lighter colours depicting a higher count of pixels (i.e. area). (F) Shows the variable importance values, ranging from 0 (no importance) to 1 (high importance), of the covariates used in the Random Forest model for predicting CWM traits, with blue colour showing importance for Senitnel-2 spectral bands, yellow for vegetation indices, green for texture metrics, maroon for climate and terrain variables and brown for soil related variables. In (A) and (B) red to orange show areas with low to intermediate trait CWM or prediction standard error respectively, while light to dark blue depicts areas with intermediate to high CWM trait values or standard error trait predictions respectively. For visualisation purposes we excluded the extreme lowest and highest 1% of values in the maps. In (C) and (D) the horizontal black line depicts the median CWM trait value and vertical lines show the whiskers extending to the largest CWM trait value or not further than 1.5 times the interquartile range and filled circles above and below the vertical line depict outlier values. For statistical model results of (A) and (B) see Supplementary Table 3. For the significance of differences between CWM trait mean values in (C) and (D), obtained using a T-test, see Supplementary Tables 4 and 5.

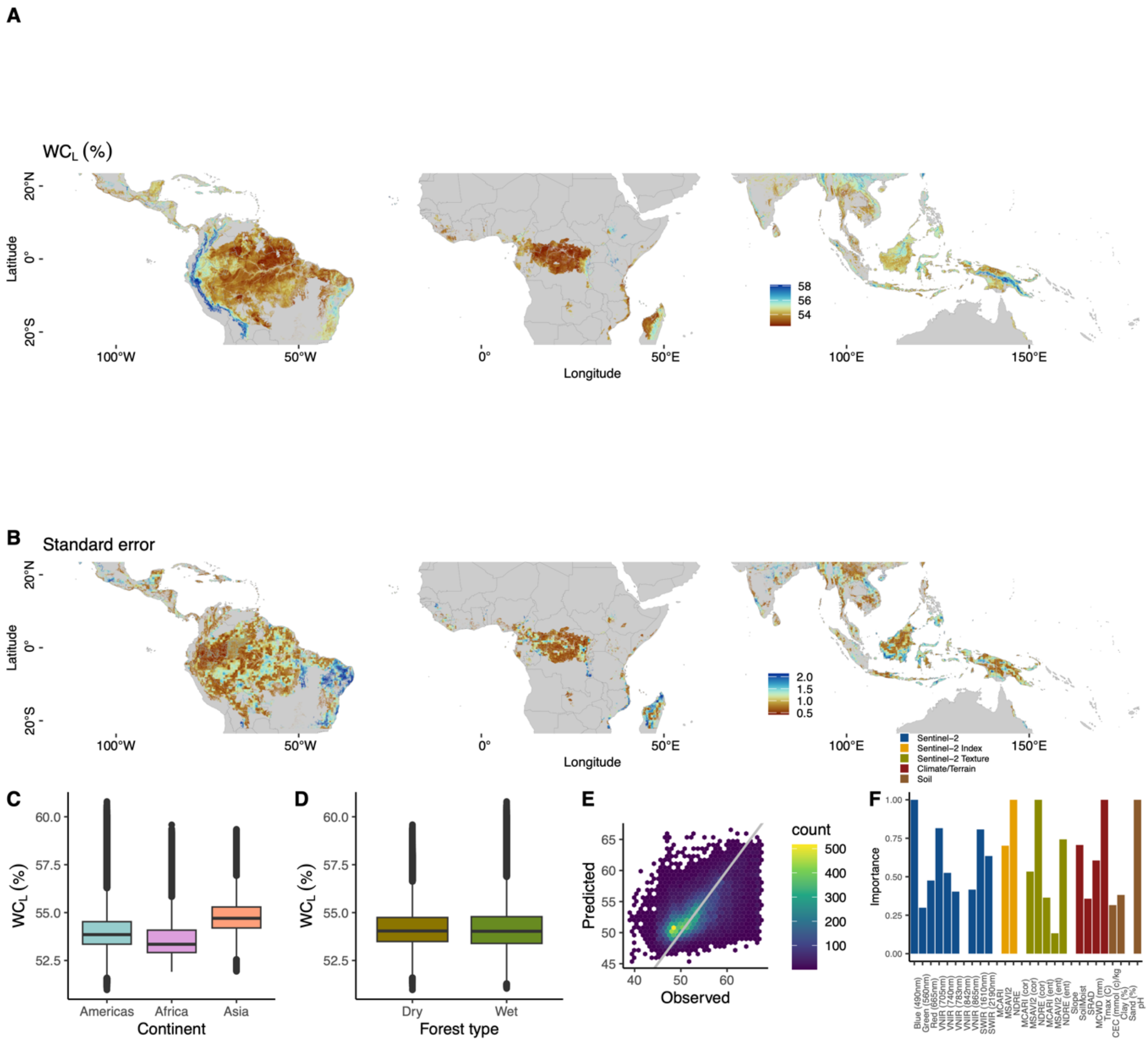

Supplementary Figure 6. Predicted distribution of community weighted mean (CWM) leaf water content (WC*L*). (A) Shows the predicted spatial distribution of this tree trait. (B) Standard Error of predicted distribution of CWM. (C) Shows boxplots for the CWM trait distribution values for tropical America, Africa and Asia extracted from the spatial predictions in (A). (D) Shows boxplots for the CWM trait distribution values for tropical dry (Dry) and tropical wet (Wet) forests across the tropical American, African and Asian forests classified and extracted from the spatial predictions in (A) using the RESOLVE Ecoregions dataset (ecoregions.appspot.com). (E) Shows the predicted versus observed CWM trait values with lighter colours depicting a higher count of pixels (i.e. area). (F) Shows the variable importance values, ranging from 0 (no importance) to 1 (high importance), of the covariates used in the Random Forest model for predicting CWM traits, with blue colour showing importance for Senitnel-2 spectral bands, yellow for vegetation indices, green for texture metrics, maroon for climate and terrain variables and brown for soil related variables. In (A) and (B) red to orange show areas with low to intermediate trait CWM or prediction standard error respectively, while light to dark blue depicts areas with intermediate to high CWM trait values or standard error trait predictions respectively. For visualisation purposes we excluded the extreme lowest and highest 1% of values in the maps. In (C) and (D) the horizontal black line depicts the median CWM trait value and vertical lines show the whiskers extending to the largest CWM trait value or not further than 1.5 times the interquartile range and filled circles above and below the vertical line depict outlier values. For statistical model results of (A) and (B) see Supplementary Table 3. For the significance of differences between CWM trait mean values in (C) and (D), obtained using a T-test, see Supplementary Tables 4 and 5.

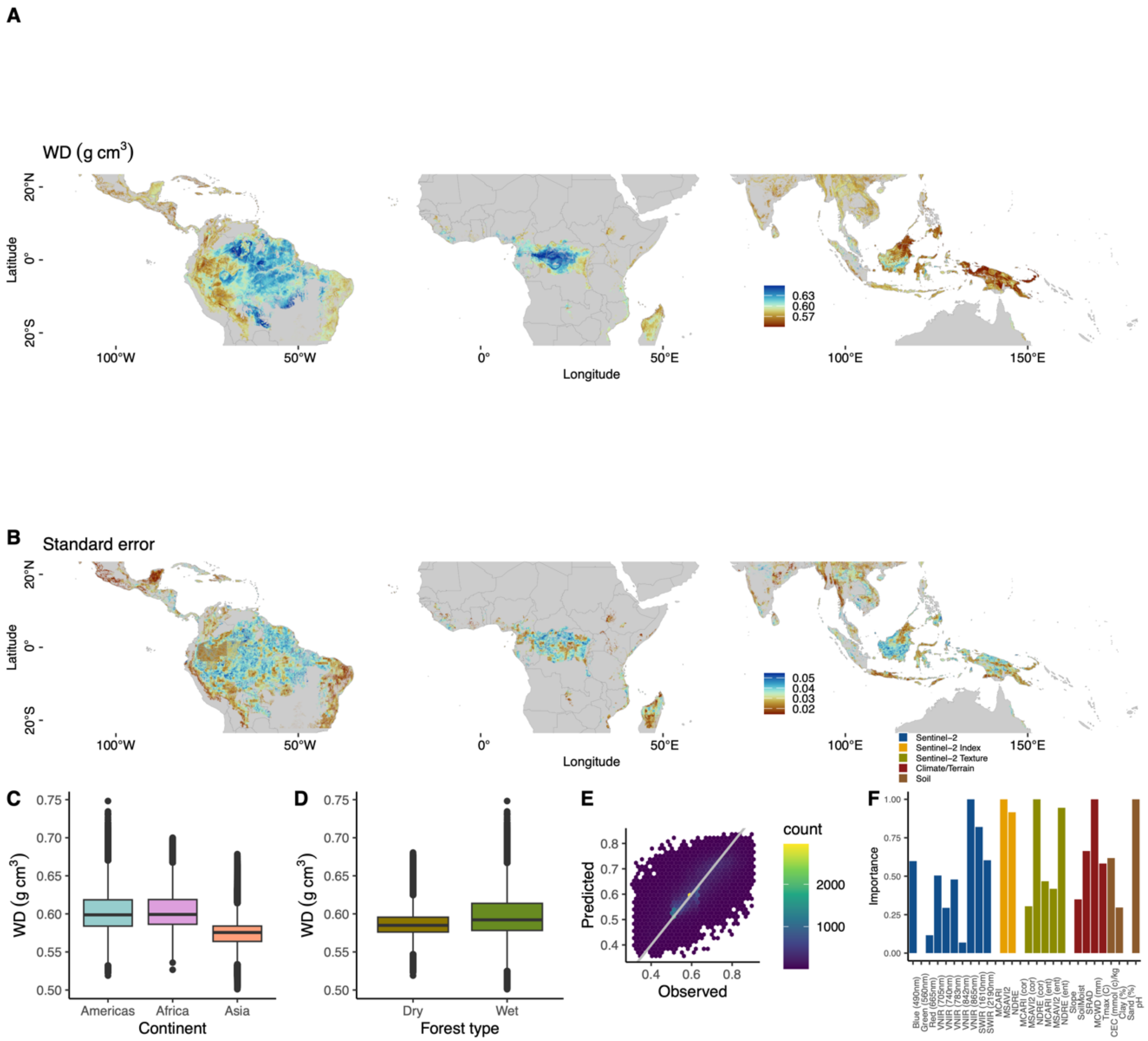

Supplementary Figure 7. Predicted distribution of community weighted mean (CWM) wood density (WD). (A) Shows the predicted spatial distribution of this tree trait. (B) Standard Error of predicted distribution of CWM. (C) Shows boxplots for the CWM trait distribution values for tropical America, Africa and Asia extracted from the spatial predictions in (A). (D) Shows boxplots for the CWM trait distribution values for tropical dry (Dry) and tropical wet (Wet) forests across the tropical American, African and Asian forests classified and extracted from the spatial predictions in (A) using the RESOLVE Ecoregions dataset (ecoregions.appspot.com). (E) Shows the predicted versus observed CWM trait values with lighter colours depicting a higher count of pixels (i.e. area). (F) Shows the variable importance values, ranging from 0 (no importance) to 1 (high importance), of the covariates used in the Random Forest model for predicting CWM traits, with blue colour showing importance for Senitnel-2 spectral bands, yellow for vegetation indices, green for texture metrics, maroon for climate and terrain variables and brown for soil related variables. In (A) and (B) red to orange show areas with low to intermediate trait CWM or prediction standard error respectively, while light to dark blue depicts areas with intermediate to high CWM trait values or standard error trait predictions respectively. For visualisation purposes we excluded the extreme lowest and highest 1% of values in the maps. In (C) and (D) the horizontal black line depicts the median CWM trait value and vertical lines show the whiskers extending to the largest CWM trait value or not further than 1.5 times the interquartile range and filled circles above and below the vertical line depict outlier values. For statistical model results of (A) and (B) see Supplementary Table 3. For the significance of differences between CWM trait mean values in (C) and (D), obtained using a T-test, see Supplementary Tables 4 and 5.

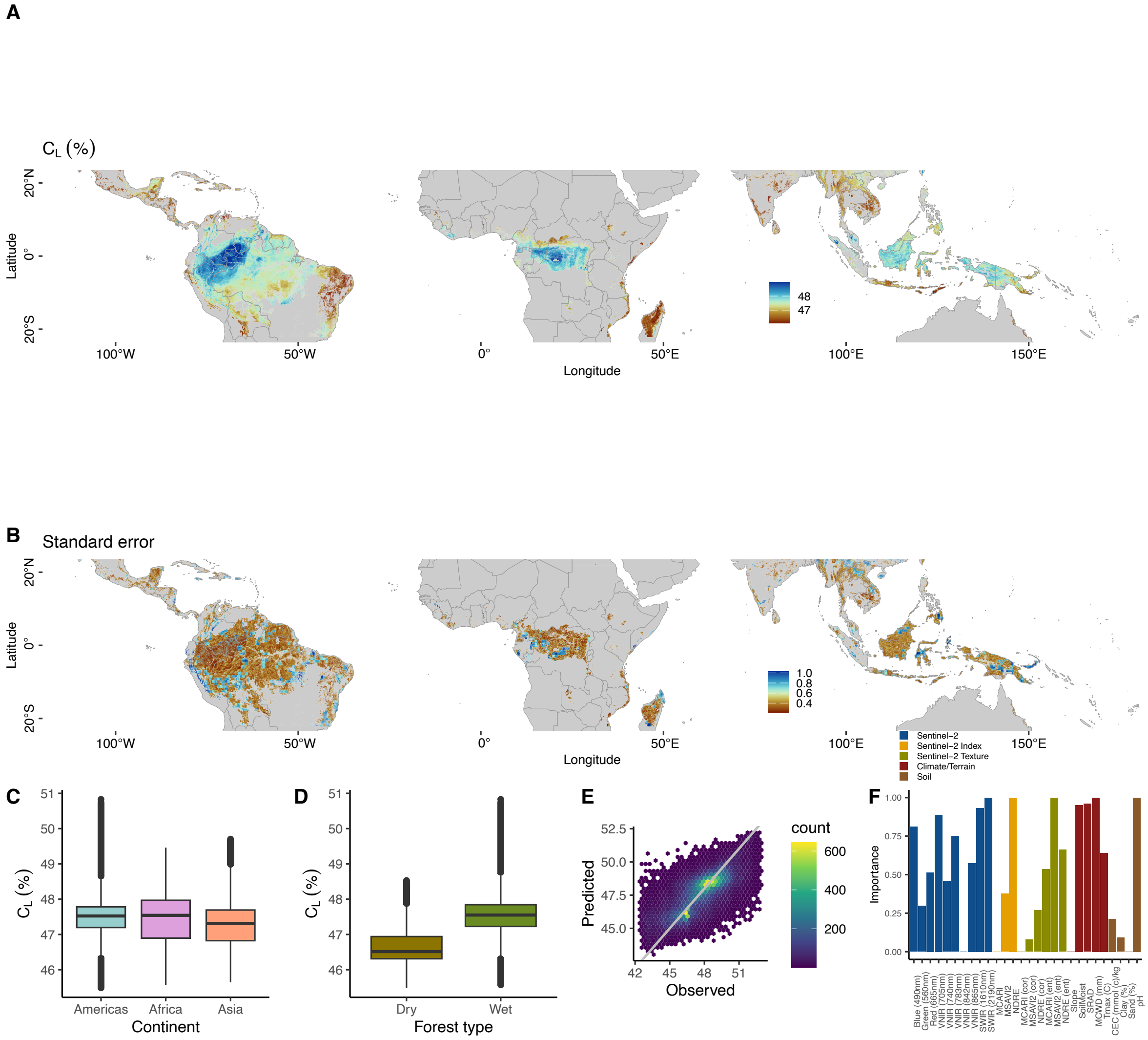

Supplementary Figure 8. Predicted distribution of community weighted mean (CWM) leaf carbon concentration (C_L). (A) Shows the predicted spatial distribution of this tree trait. (B) Standard Error of predicted distribution of CWM. (C) Shows boxplots for the CWM trait distribution values for tropical America, Africa and Asia extracted from the spatial predictions in (A). (D) Shows boxplots for the CWM trait distribution values for tropical dry (Dry) and tropical wet (Wet) forests across the tropical American, African and Asian forests classified and extracted from the spatial predictions in (A) using the RESOLVE Ecoregions dataset (ecoregions.appspot.com). (E) Shows the predicted versus observed CWM trait values with lighter colours depicting a higher count of pixels (i.e. area). (F) Shows the variable importance values, ranging from 0 (no importance) to 1 (high importance), of the covariates used in the Random Forest model for predicting CWM traits, with blue colour showing importance for Senitnel-2 spectral bands, yellow for vegetation indices, green for texture metrics, maroon for climate and terrain variables and brown for soil related variables. In (A) and (B) red to orange show areas with low to intermediate trait CWM or prediction standard error respectively, while light to dark blue depicts areas with intermediate to high CWM trait values or standard error trait predictions respectively. For visualisation purposes we excluded the extreme lowest and highest 1% of values in the maps. In (C) and (D) the horizontal black line depicts the median CWM trait value and vertical lines show the whiskers extending to the largest CWM trait value or not further than 1.5 times the interquartile range and filled circles above and below the vertical line depict outlier values. For statistical model results of (A) and (B) see Supplementary Table 3. For the significance of differences between CWM trait mean values in (C) and (D), obtained using a T-test, see Supplementary Tables 4 and 5.

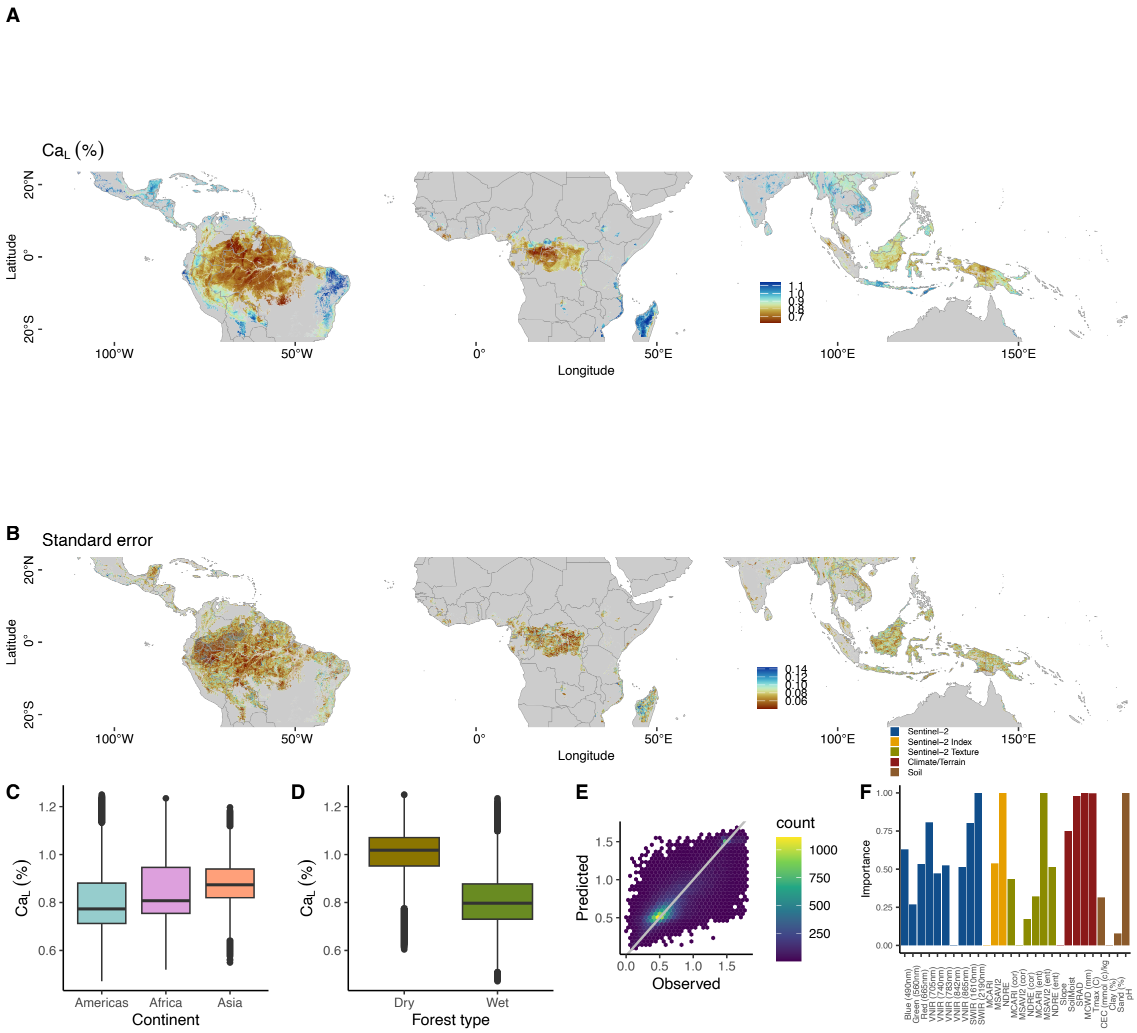

Supplementary Figure 9. Predicted distribution of community weighted mean (CWM) leaf calcium concentration (Ca*$_L$*). (A) Shows the predicted spatial distribution of this tree trait. (B) Standard Error of predicted distribution of CWM. (C) Shows boxplots for the CWM trait distribution values for tropical America, Africa and Asia extracted from the spatial predictions in (A). (D) Shows boxplots for the CWM trait distribution values for tropical dry (Dry) and tropical wet (Wet) forests across the tropical American, African and Asian forests classified and extracted from the spatial predictions in (A) using the RESOLVE Ecoregions dataset (ecoregions.appspot.com). (E) Shows the predicted versus observed CWM trait values with lighter colours depicting a higher count of pixels (i.e. area). (F) Shows the variable importance values, ranging from 0 (no importance) to 1 (high importance), of the covariates used in the Random Forest model for predicting CWM traits, with blue colour showing importance for Senitnel-2 spectral bands, yellow for vegetation indices, green for texture metrics, maroon for climate and terrain variables and brown for soil related variables. In (A) and (B) red to orange show areas with low to intermediate trait CWM or prediction standard error respectively, while light to dark blue depicts areas with intermediate to high CWM trait values or standard error trait predictions respectively. For visualisation purposes we excluded the extreme lowest and highest 1% of values in the maps. In (C) and (D) the horizontal black line depicts the median CWM trait value and vertical lines show the whiskers extending to the largest CWM trait value or not further than 1.5 times the interquartile range and filled circles above and below the vertical line depict outlier values. For statistical model results of (A) and (B) see Supplementary Table 3. For the significance of differences between CWM trait mean values in (C) and (D), obtained using a T-test, see Supplementary Tables 4 and 5.

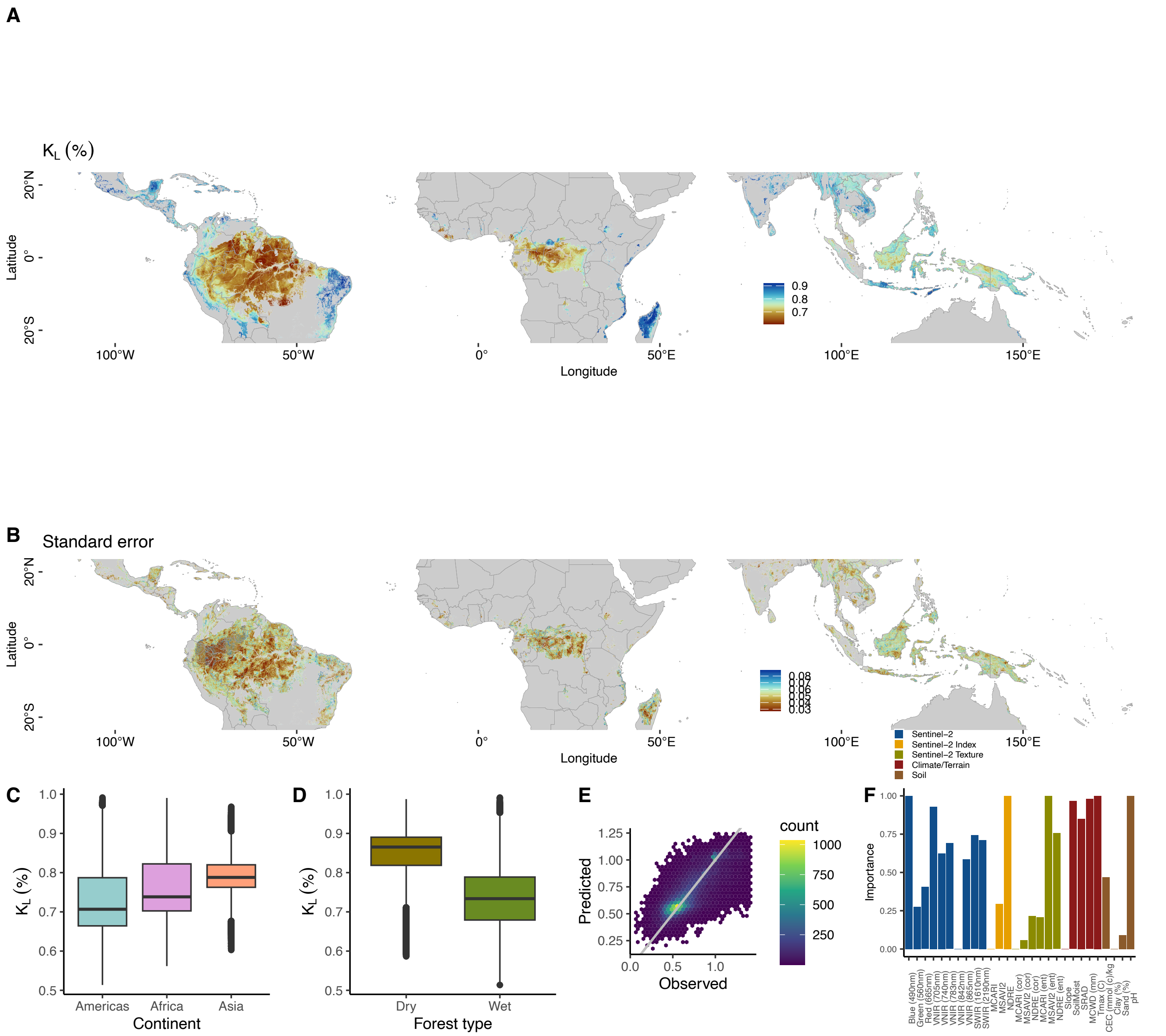

Supplementary Figure 10. Predicted distribution of community weighted mean (CWM) leaf potassium concentration (K_L). (A) Shows the predicted spatial distribution of this tree trait. (B) Standard Error of predicted distribution of CWM. (C) Shows boxplots for the CWM trait distribution values for tropical America, Africa and Asia extracted from the spatial predictions in (A). (D) Shows boxplots for the CWM trait distribution values for tropical dry (Dry) and tropical wet (Wet) forests across the tropical American, African and Asian forests classified and extracted from the spatial predictions in (A) using the RESOLVE Ecoregions dataset (ecoregions.appspot.com). (E) Shows the predicted versus observed CWM trait values with lighter colours depicting a higher count of pixels (i.e. area). (F) Shows the variable importance values, ranging from 0 (no importance) to 1 (high importance), of the covariates used in the Random Forest model for predicting CWM traits, with blue colour showing importance for Senitnel-2 spectral bands, yellow for vegetation indices, green for texture metrics, maroon for climate and terrain variables and brown for soil related variables. In (A) and (B) red to orange show areas with low to intermediate trait CWM or prediction standard error respectively, while light to dark blue depicts areas with intermediate to high CWM trait values or standard error trait predictions respectively. For visualisation purposes we excluded the extreme lowest and highest 1% of values in the maps. In (C) and (D) the horizontal black line depicts the median CWM trait value and vertical lines show the whiskers extending to the largest CWM trait value or not further than 1.5 times the interquartile range and filled circles above and below the vertical line depict outlier values. For statistical model results of (A) and (B) see Supplementary Table 3. For the significance of differences between CWM trait mean values in (C) and (D), obtained using a T-test, see Supplementary Tables 4 and 5.

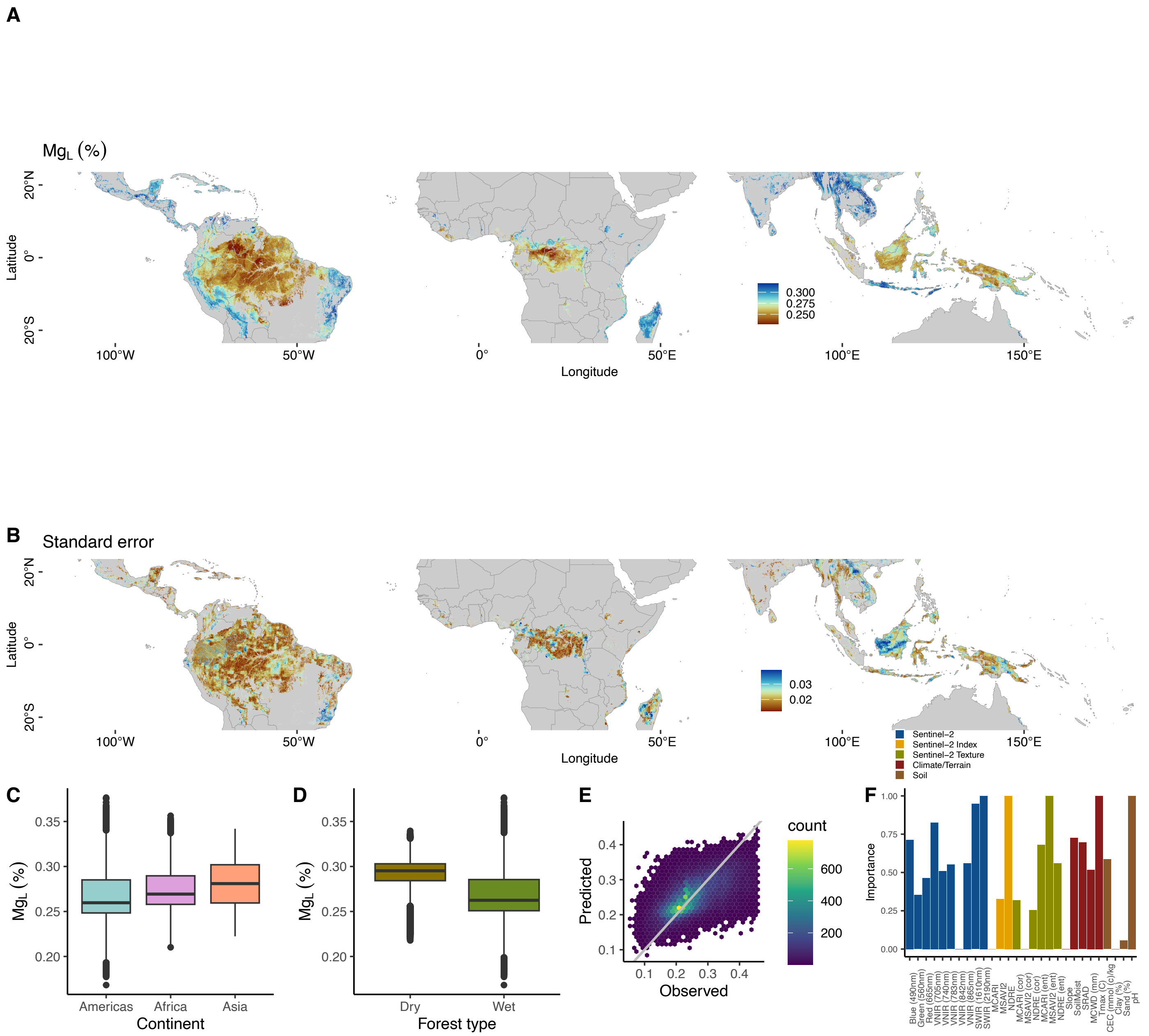

Supplementary Figure 11. Predicted distribution of community weighted mean (CWM) leaf magnesium concentration (Mg_L). (A) Shows the predicted spatial distribution of this tree trait. (B) Standard Error of predicted distribution of CWM. (C) Shows boxplots for the CWM trait distribution values for tropical America, Africa and Asia extracted from the spatial predictions in (A). (D) Shows boxplots for the CWM trait distribution values for tropical dry (Dry) and tropical wet (Wet) forests across the tropical American, African and Asian forests classified and extracted from the spatial predictions in (A) using the RESOLVE Ecoregions dataset (ecoregions.appspot.com). (E) Shows the predicted versus observed CWM trait values with lighter colours depicting a higher count of pixels (i.e. area). (F) Shows the variable importance values, ranging from 0 (no importance) to 1 (high importance), of the covariates used in the Random Forest model for predicting CWM traits, with blue colour showing importance for Senitnel-2 spectral bands, yellow for vegetation indices, green for texture metrics, maroon for climate and terrain variables and brown for soil related variables. In (A) and (B) red to orange show areas with low to intermediate trait CWM or prediction standard error respectively, while light to dark blue depicts areas with intermediate to high CWM trait values or standard error trait predictions respectively. For visualisation purposes we excluded the extreme lowest and highest 1% of values in the maps. In (C) and (D) the horizontal black line depicts the median CWM trait value and vertical lines show the whiskers extending to the largest CWM trait value or not further than 1.5 times the interquartile range and filled circles above and below the vertical line depict outlier values. For statistical model results of (A) and (B) see Supplementary Table 3. For the significance of differences between CWM trait mean values in (C) and (D), obtained using a T-test, see Supplementary Tables 4 and 5.

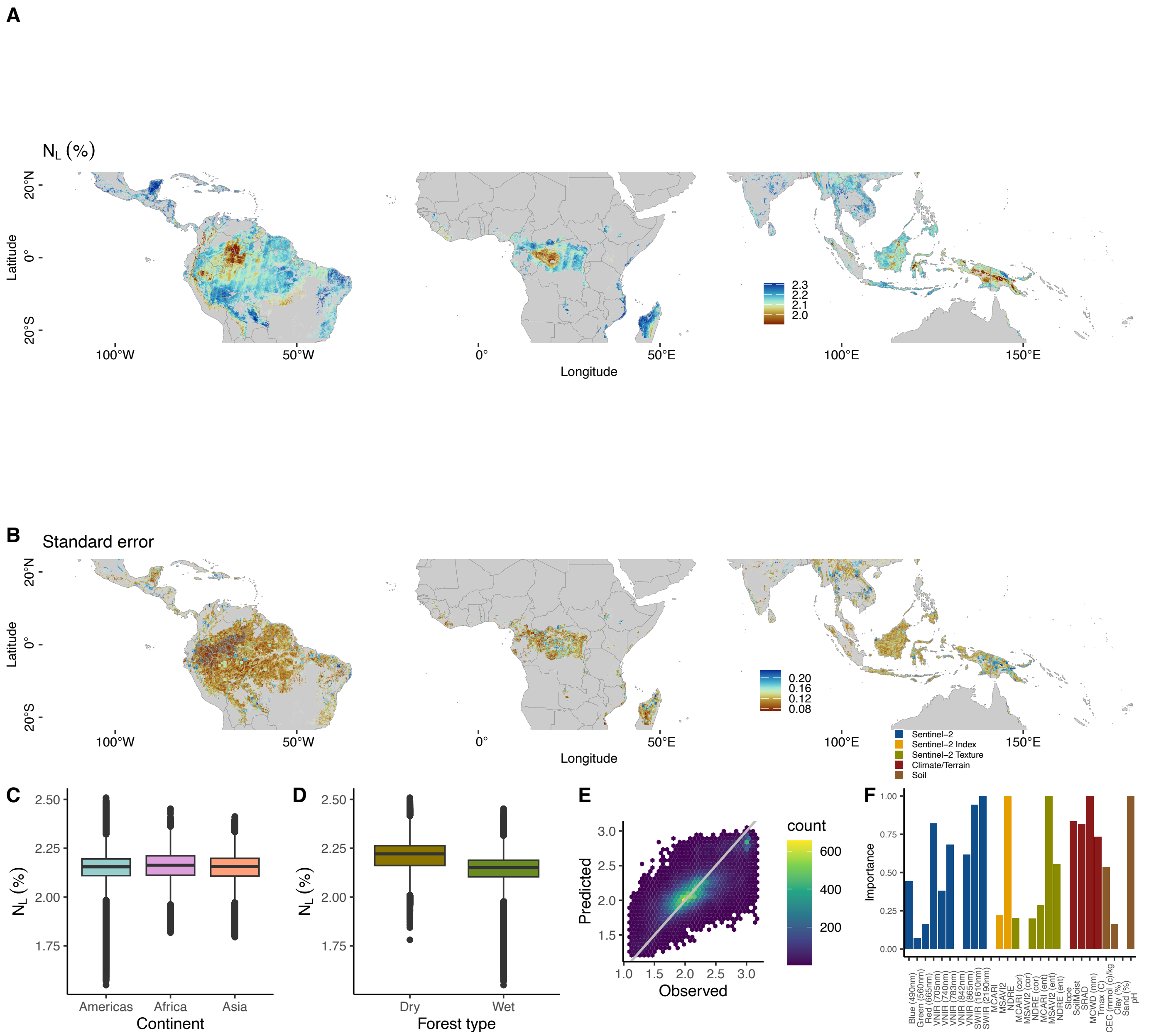

Supplementary Figure 12. Predicted distribution of community weighted mean (CWM) leaf nitrogen concentration (N_L). (A) Shows the predicted spatial distribution of this tree trait. (B) Standard Error of predicted distribution of CWM. (C) Shows boxplots for the CWM trait distribution values for tropical America, Africa and Asia extracted from the spatial predictions in (A). (D) Shows boxplots for the CWM trait distribution values for tropical dry (Dry) and tropical wet (Wet) forests across the tropical American, African and Asian forests classified and extracted from the spatial predictions in (A) using the RESOLVE Ecoregions dataset (ecoregions.appspot.com). (E) Shows the predicted versus observed CWM trait values with lighter colours depicting a higher count of pixels (i.e. area). (F) Shows the variable importance values, ranging from 0 (no importance) to 1 (high importance), of the covariates used in the Random Forest model for predicting CWM traits, with blue colour showing importance for Senitnel-2 spectral bands, yellow for vegetation indices, green for texture metrics, maroon for climate and terrain variables and brown for soil related variables. In (A) and (B) red to orange show areas with low to intermediate trait CWM or prediction standard error respectively, while light to dark blue depicts areas with intermediate to high CWM trait values or standard error trait predictions respectively. For visualisation purposes we excluded the extreme lowest and highest 1% of values in the maps. In (C) and (D) the horizontal black line depicts the median CWM trait value and vertical lines show the whiskers extending to the largest CWM trait value or not further than 1.5 times the interquartile range and filled circles above and below the vertical line depict outlier values. For statistical model results of (A) and (B) see Supplementary Table 3. For the significance of differences between CWM trait mean values in (C) and (D), obtained using a T-test, see Supplementary Tables 4 and 5.

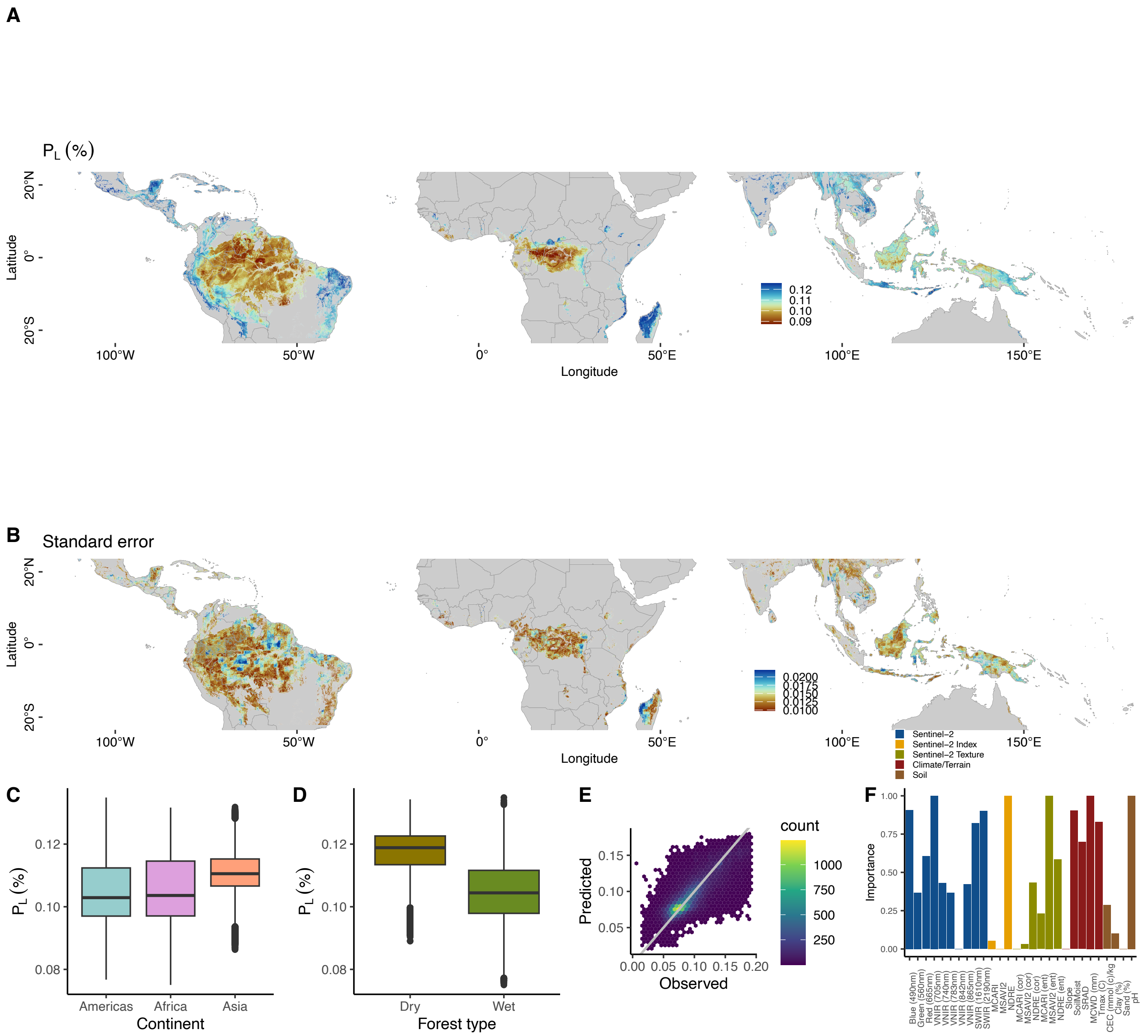

Supplementary Figure 13. Predicted distribution of community weighted mean (CWM) leaf phosphorus concentration (P_L). (A) Shows the predicted spatial distribution of this tree trait. (B) Standard Error of predicted distribution of CWM. (C) Shows boxplots for the CWM trait distribution values for tropical America, Africa and Asia extracted from the spatial predictions in (A). (D) Shows boxplots for the CWM trait distribution values for tropical dry (Dry) and tropical wet (Wet) forests across the tropical American, African and Asian forests classified and extracted from the spatial predictions in (A) using the RESOLVE Ecoregions dataset (ecoregions.appspot.com). (E) Shows the predicted versus observed CWM trait values with lighter colours depicting a higher count of pixels (i.e. area). (F) Shows the variable importance values, ranging from 0 (no importance) to 1 (high importance), of the covariates used in the Random Forest model for predicting CWM traits, with blue colour showing importance for Senitnel-2 spectral bands, yellow for vegetation indices, green for texture metrics, maroon for climate and terrain variables and brown for soil related variables. In (A) and (B) red to orange show areas with low to intermediate trait CWM or prediction standard error respectively, while light to dark blue depicts areas with intermediate to high CWM trait values or standard error trait predictions respectively. For visualisation purposes we excluded the extreme lowest and highest 1% of values in the maps. In (C) and (D) the horizontal black line depicts the median CWM trait value and vertical lines show the whiskers extending to the largest CWM trait value or not further than 1.5 times the interquartile range and filled circles above and below the vertical line depict outlier values. For statistical model results of (A) and (B) see Supplementary Table 3. For the significance of differences between CWM trait mean values in (C) and (D), obtained using a T-test, see Supplementary Tables 4 and 5.

Table S1. Model accuracy results for each of the traits modelled using the spatial block cross-validation approach. R2: variance explained, RMSE: root mean squared error, MSE: mean squared error, MAE: mean absolute error. Area: leaf area, C: leaf carbon concentration, Ca: leaf calcium concentration, DM: leaf dry mass, FM: leaf fresh mass, K: leaf potasium concentration, Mg: leaf magnesium concentration, N: leaf nitrogen concentration, P: leaf phosphorus concentration, SLA: specific leaf area, Thickness: leaf thickness, WC: leaf water content, WD: wood density.

Trait	Statistic	Value	Method	Continent
WD	R2	0.48	Spatial plot out Cross-Validation	ALL
	RMSE	0.07		
	MSE	0.01		
	MAE	0.05		
C	R2	0.40		
	RMSE	1.42		
	MSE	2.03		
	MAE	1.09		
N	R2	0.53		
	RMSE	0.29		
	MSE	0.09		
	MAE	0.23		
P	R2	0.50		
	RMSE	0.02		
	MSE	0.00		
	MAE	0.02		
Area	R2	0.22		
	RMSE	66.15		
	MSE	4375.24		
	MAE	50.18		
Ca	R2	0.64		
	RMSE	0.22		
	MSE	0.05		
	MAE	0.15		
K	R2	0.46		
	RMSE	0.17		
	MSE	0.03		
	MAE	0.13		
DM	R2	0.32		
	RMSE	0.58		
	MSE	0.33		
	MAE	0.43		
Mg	R2	0.27		

	RMSE	0.06		
	MSE	0.00		
	MAE	0.05		
SLA	R2	0.32		
	RMSE	19.95		
	MSE	398.03		
	MAE	15.52		
Thickness	R2	0.17		
	RMSE	0.79		
	MSE	0.62		
	MAE	0.52		
FM	R2	0.31		
	RMSE	2.24		
	MSE	5.02		
	MAE	1.66		
WC	R2	0.18		
	RMSE	3.92		
	MSE	15.37		
	MAE	3.03		
WD	R2	0.23	Spatial plot out Cross-Validation	Africa
	RMSE	0.10		
	MSE	0.01		
	MAE	0.08		
C	R2	0.62		
	RMSE	1.42		
	MSE	2.02		
	MAE	1.08		
N	R2	0.57		
	RMSE	0.32		
	MSE	0.10		
	MAE	0.26		
P	R2	0.44		
	RMSE	0.03		
	MSE	0.00		
	MAE	0.02		
Area	R2	0.14		
	RMSE	72.72		
	MSE	5287.85		
	MAE	59.08		
Ca	R2	0.80		
	RMSE	0.21		
	MSE	0.04		
	MAE	0.14		
K	R2	0.33		

	RMSE	0.14		
	MSE	0.02		
	MAE	0.10		
DM	R2	0.14		
	RMSE	0.86		
	MSE	0.74		
	MAE	0.73		
Mg	R2	0.10		
	RMSE	0.07		
	MSE	0.00		
	MAE	0.06		
SLA	R2	0.26		
	RMSE	20.80		
	MSE	432.55		
	MAE	17.10		
Thickness	R2	0.18		
	RMSE	0.31		
	MSE	0.10		
	MAE	0.15		
FM	R2	0.13		
	RMSE	2.79		
	MSE	7.76		
	MAE	2.33		
WC	R2	0.13		
	RMSE	4.60		
	MSE	21.14		
	MAE	3.47		
WD	R2	0.22		
	RMSE	0.09		
	MSE	0.01		
	MAE	0.07		
C	R2	0.00		
	RMSE	1.66		
	MSE	2.76		
	MAE	1.34		
N	R2	0.28	Spatial plot out Cross-Validation	Asia
	RMSE	0.34		
	MSE	0.11		
	MAE	0.27		
P	R2	0.32		
	RMSE	0.03		
	MSE	0.00		
	MAE	0.02		
Area	R2	0.28		

	RMSE	66.41
	MSE	4410.25
	MAE	51.88
Ca	R2	0.01
	RMSE	0.37
	MSE	0.14
	MAE	0.30
K	R2	0.11
	RMSE	0.21
	MSE	0.05
	MAE	0.17
DM	R2	0.01
	RMSE	0.67
	MSE	0.45
	MAE	0.53
Mg	R2	0.02
	RMSE	0.08
	MSE	0.01
	MAE	0.07
SLA	R2	0.26
	RMSE	20.86
	MSE	435.21
	MAE	16.24
Thickness	R2	0.02
	RMSE	0.18
	MSE	0.03
	MAE	0.13
FM	R2	0.003
	RMSE	2.99
	MSE	8.94
	MAE	2.25
WC	R2	0.002
	RMSE	4.52
	MSE	20.47
	MAE	3.56

Table S2. Results of the t-test analysis, with Bonferroni correction, testing for significance of differences between the community level trait values among continents. Area: leaf area, C: leaf carbon concentration, Ca: leaf calcium concentration, FM: leaf fresh mass, DM: leaf dry mass, K: leaf potassium concentration, Mg: leaf magnesium concentration, N: leaf nitrogen concentration, P: leaf phosphorus concentration, SLA: specific leaf area, Thickness: leaf thickness, WC: leaf water content, WD: wood density. The dataset specifies if the full (Full) number of pixels from the predictions were used or a random 10% or 1% of them to test the effect of sample size on the t-test results. Group: continent being compared, Mean value: average community weight trait value, value difference: difference between average community weighted mean trait value from group 1 and group 2. Conf-low: lower confidence interval, Conf-High: highest confidence interval, Statistic: t-test statistic value, n1= sample size for group 1, n2=sample size for group 2, P-val adj.: adjusted P-value. Non statistically significant comparisons are highlighted in grey.

Dataset	Trait	Group 1	Group 2	Mean value 1	Mean value 2	Value difference	Conf-low	Conf-High	Statistic	n1	n2	P-val adj.
Full	Area	Africa	Americas	101.41	114.39	-12.98	-13.01	-12.95	-969.75	9.1E+05	3.2E+06	< 0.0001
	Area	Africa	Asia	101.41	119.30	-17.88	-17.92	-17.85	-1045.72	9.1E+05	1.4E+06	< 0.0001
	Area	Americas	Asia	114.39	119.30	-4.90	-4.94	-4.87	-310.71	3.2E+06	1.4E+06	< 0.0001
	C	Africa	Americas	47.45	47.48	-0.03	-0.04	-0.03	-43.58	9.1E+05	3.2E+06	< 0.0001
	C	Africa	Asia	47.45	47.26	0.19	0.18	0.19	223.63	9.1E+05	1.4E+06	< 0.0001
	C	Americas	Asia	47.48	47.26	0.22	0.22	0.22	398.89	3.2E+06	1.4E+06	< 0.0001
	Ca	Africa	Americas	0.85	0.81	0.04	0.04	0.04	256.53	9.0E+05	3.2E+06	< 0.0001
	Ca	Africa	Asia	0.85	0.88	-0.04	-0.04	-0.04	-243.14	9.0E+05	1.5E+06	< 0.0001
	Ca	Americas	Asia	0.81	0.88	-0.08	-0.08	-0.08	-766.55	3.2E+06	1.5E+06	< 0.0001
	DM	Africa	Americas	0.94	0.99	-0.05	-0.05	-0.05	-396.03	9.2E+05	3.2E+06	< 0.0001
	DM	Africa	Asia	0.94	1.06	-0.12	-0.12	-0.12	-800.83	9.2E+05	1.4E+06	< 0.0001
	DM	Americas	Asia	0.99	1.06	-0.07	-0.07	-0.07	-599.23	3.2E+06	1.4E+06	< 0.0001
	FM	Africa	Americas	3.20	3.40	-0.21	-0.21	-0.21	-453.91	9.0E+05	3.2E+06	< 0.0001
	FM	Africa	Asia	3.20	3.90	-0.70	-0.70	-0.70	-1317.34	9.0E+05	1.4E+06	< 0.0001
	FM	Americas	Asia	3.40	3.90	-0.49	-0.49	-0.49	-1263.15	3.2E+06	1.4E+06	< 0.0001
	K	Africa	Americas	0.76	0.73	0.03	0.03	0.03	306.59	9.0E+05	3.2E+06	< 0.0001
	K	Africa	Asia	0.76	0.79	-0.04	-0.04	-0.04	-394.40	9.0E+05	1.5E+06	< 0.0001
	K	Americas	Asia	0.73	0.79	-0.06	-0.06	-0.06	-1083.03	3.2E+06	1.5E+06	< 0.0001
	Mg	Africa	Americas	0.27	0.27	0.01	0.01	0.01	265.64	9.0E+05	3.2E+06	< 0.0001
	Mg	Africa	Asia	0.27	0.28	-0.01	-0.01	-0.01	-265.95	9.0E+05	1.4E+06	< 0.0001

	Mg	Americas	Asia	0.27	0.28	-0.01	-0.01	-0.01	-617.76	3.2E+06	1.4E+06	< 0.0001
	N	Africa	Americas	2.16	2.15	0.01	0.01	0.01	64.64	9.1E+05	3.2E+06	< 0.0001
	N	Africa	Asia	2.16	2.15	0.01	0.01	0.01	52.82	9.1E+05	1.4E+06	< 0.0001
	N	Americas	Asia	2.15	2.15	0.00	0.00	0.00	-6.68	3.2E+06	1.4E+06	7.32E-11
	P	Africa	Americas	0.11	0.10	0.00	0.00	0.00	90.93	8.8E+05	3.2E+06	< 0.0001
	P	Africa	Asia	0.11	0.11	-0.01	-0.01	-0.01	-412.87	8.8E+05	1.4E+06	< 0.0001
	P	Americas	Asia	0.10	0.11	-0.01	-0.01	-0.01	-831.01	3.2E+06	1.4E+06	< 0.0001
	SLA	Africa	Americas	133.96	131.98	1.98	1.97	1.99	345.15	9.1E+05	3.2E+06	< 0.0001
	SLA	Africa	Asia	133.96	130.53	3.43	3.42	3.44	503.37	9.1E+05	1.4E+06	< 0.0001
	SLA	Americas	Asia	131.98	130.53	1.45	1.44	1.46	266.34	3.2E+06	1.4E+06	< 0.0001
	Thickness	Africa	Americas	0.85	0.82	0.04	0.04	0.04	278.68	9.0E+05	3.2E+06	< 0.0001
	Thickness	Africa	Asia	0.85	0.80	0.06	0.06	0.06	420.88	9.0E+05	1.4E+06	< 0.0001
	Thickness	Americas	Asia	0.82	0.80	0.02	0.02	0.02	218.25	3.2E+06	1.4E+06	< 0.0001
	WC	Africa	Americas	53.64	54.04	-0.40	-0.41	-0.40	-359.63	9.0E+05	3.2E+06	< 0.0001
	WC	Africa	Asia	53.64	54.80	-1.17	-1.17	-1.16	-972.10	9.0E+05	1.5E+06	< 0.0001
	WC	Americas	Asia	54.04	54.80	-0.76	-0.76	-0.76	-852.02	3.2E+06	1.5E+06	< 0.0001
	WD	Africa	Americas	0.60	0.60	0.00	0.00	0.00	78.55	9.0E+05	3.2E+06	< 0.0001
	WD	Africa	Asia	0.60	0.58	0.03	0.03	0.03	1027.35	9.0E+05	1.4E+06	< 0.0001
	WD	Americas	Asia	0.60	0.58	0.03	0.03	0.03	1394.24	3.2E+06	1.4E+06	< 0.0001
	Area	Africa	Americas	101.41	114.45	-13.04	-13.12	-12.96	-307.67	9.1E+04	3.2E+05	< 0.0001
	Area	Africa	Asia	101.41	119.26	-17.84	-17.95	-17.74	-329.63	9.1E+04	1.4E+05	< 0.0001
	Area	Americas	Asia	114.45	119.26	-4.81	-4.90	-4.71	-96.32	3.2E+05	1.4E+05	< 0.0001
	C	Africa	Americas	47.44	47.48	-0.04	-0.04	-0.03	-14.78	9.1E+04	3.2E+05	7E-49
**10%**	C	Africa	Asia	47.44	47.26	0.18	0.18	0.19	70.05	9.1E+04	1.4E+05	< 0.0001
	C	Americas	Asia	47.48	47.26	0.22	0.22	0.22	126.75	3.2E+05	1.4E+05	< 0.0001
	Ca	Africa	Americas	0.85	0.81	0.04	0.04	0.04	82.26	9.0E+04	3.2E+05	< 0.0001
	Ca	Africa	Asia	0.85	0.88	-0.04	-0.04	-0.04	-75.58	9.0E+04	1.5E+05	< 0.0001
	Ca	Americas	Asia	0.81	0.88	-0.08	-0.08	-0.07	-242.77	3.2E+05	1.5E+05	< 0.0001
	DM	Africa	Americas	0.94	0.99	-0.05	-0.05	-0.05	-124.33	9.2E+04	3.2E+05	< 0.0001

DM	Africa	Asia	0.94	1.06	-0.12	-0.12	-0.12	-253.28	9.2E+04	1.4E+05	< 0.0001
DM	Americas	Asia	0.99	1.06	-0.07	-0.07	-0.07	-190.32	3.2E+05	1.4E+05	< 0.0001
FM	Africa	Americas	3.20	3.41	-0.21	-0.21	-0.21	-144.89	9.0E+04	3.2E+05	< 0.0001
FM	Africa	Asia	3.20	3.90	-0.70	-0.70	-0.69	-416.78	9.0E+04	1.4E+05	< 0.0001
FM	Americas	Asia	3.41	3.90	-0.49	-0.49	-0.49	-397.57	3.2E+05	1.4E+05	< 0.0001
K	Africa	Americas	0.76	0.73	0.03	0.03	0.03	97.28	9.0E+04	3.2E+05	< 0.0001
K	Africa	Asia	0.76	0.79	-0.04	-0.04	-0.04	-125.94	9.0E+04	1.5E+05	< 0.0001
K	Americas	Asia	0.73	0.79	-0.06	-0.06	-0.06	-344.41	3.2E+05	1.5E+05	< 0.0001
Mg	Africa	Americas	0.27	0.27	0.01	0.01	0.01	84.91	9.0E+04	3.2E+05	< 0.0001
Mg	Africa	Asia	0.27	0.28	-0.01	-0.01	-0.01	-85.29	9.0E+04	1.4E+05	< 0.0001
Mg	Americas	Asia	0.27	0.28	-0.01	-0.01	-0.01	-197.75	3.2E+05	1.4E+05	< 0.0001
N	Africa	Americas	2.16	2.15	0.01	0.01	0.01	20.23	9.1E+04	3.2E+05	2E-90
N	Africa	Asia	2.16	2.15	0.01	0.01	0.01	17.47	9.1E+04	1.4E+05	8E-68
N	Americas	Asia	2.15	2.15	0.00	0.00	0.00	-0.77	3.2E+05	1.4E+05	1
P	Africa	Americas	0.11	0.10	0.00	0.00	0.00	27.70	8.8E+04	3.2E+05	6E-168
P	Africa	Asia	0.11	0.11	-0.01	-0.01	0.00	-131.57	8.8E+04	1.4E+05	< 0.0001
P	Americas	Asia	0.10	0.11	-0.01	-0.01	-0.01	-262.79	3.2E+05	1.4E+05	< 0.0001
SLA	Africa	Americas	133.95	131.97	1.98	1.95	2.02	109.06	9.1E+04	3.2E+05	< 0.0001
SLA	Africa	Asia	133.95	130.54	3.41	3.37	3.45	158.14	9.1E+04	1.4E+05	< 0.0001
SLA	Americas	Asia	131.97	130.54	1.42	1.39	1.46	82.83	3.2E+05	1.4E+05	< 0.0001
Thickness	Africa	Americas	0.85	0.82	0.04	0.03	0.04	88.58	9.0E+04	3.2E+05	< 0.0001
Thickness	Africa	Asia	0.85	0.80	0.06	0.06	0.06	132.53	9.0E+04	1.4E+05	< 0.0001
Thickness	Americas	Asia	0.82	0.80	0.02	0.02	0.02	67.85	3.2E+05	1.4E+05	< 0.0001
WC	Africa	Americas	53.64	54.04	-0.40	-0.41	-0.40	-114.00	9.0E+04	3.2E+05	< 0.0001
WC	Africa	Asia	53.64	54.80	-1.17	-1.18	-1.16	-308.00	9.0E+04	1.5E+05	< 0.0001
WC	Americas	Asia	54.04	54.80	-0.76	-0.77	-0.76	-270.03	3.2E+05	1.5E+05	< 0.0001
WD	Africa	Americas	0.60	0.60	0.00	0.00	0.00	24.48	9.0E+04	3.2E+05	1E-131
WD	Africa	Asia	0.60	0.58	0.03	0.03	0.03	324.41	9.0E+04	1.4E+05	< 0.0001
WD	Americas	Asia	0.60	0.58	0.03	0.03	0.03	440.23	3.2E+05	1.4E+05	< 0.0001

	Area	Africa	Americas	101.21	114.35	-13.14	-13.40	-12.87	-98.13	9.1E+03	3.2E+04	< 0.0001
	Area	Africa	Asia	101.21	119.13	-17.92	-18.26	-17.59	-105.39	9.1E+03	1.4E+04	< 0.0001
	Area	Americas	Asia	114.35	119.13	-4.79	-5.10	-4.48	-30.52	3.2E+04	1.4E+04	2E-200
	C	Africa	Americas	47.44	47.48	-0.04	-0.05	-0.02	-4.72	9.1E+03	3.2E+04	7E-06
	C	Africa	Asia	47.44	47.26	0.19	0.17	0.20	22.45	9.1E+03	1.4E+04	2E-109
	C	Americas	Asia	47.48	47.26	0.22	0.21	0.23	40.62	3.2E+04	1.4E+04	< 0.0001
	Ca	Africa	Americas	0.85	0.81	0.04	0.04	0.04	25.45	9.0E+03	3.2E+04	3E-139
	Ca	Africa	Asia	0.85	0.88	-0.04	-0.04	-0.03	-23.89	9.0E+03	1.5E+04	3E-123
	Ca	Americas	Asia	0.81	0.88	-0.07	-0.08	-0.07	-75.53	3.2E+04	1.5E+04	< 0.0001
	DM	Africa	Americas	0.94	0.99	-0.05	-0.05	-0.05	-39.13	9.2E+03	3.2E+04	2.31e-318
	DM	Africa	Asia	0.94	1.06	-0.12	-0.12	-0.12	-78.92	9.2E+03	1.4E+04	< 0.0001
	DM	Americas	Asia	0.99	1.06	-0.07	-0.07	-0.07	-58.91	3.2E+04	1.4E+04	< 0.0001
	FM	Africa	Americas	3.20	3.40	-0.20	-0.21	-0.19	-44.00	9.0E+03	3.2E+04	< 0.0001
	FM	Africa	Asia	3.20	3.90	-0.70	-0.71	-0.69	-131.94	9.0E+03	1.4E+04	< 0.0001
**1%**	FM	Americas	Asia	3.40	3.90	-0.50	-0.51	-0.49	-128.37	3.2E+04	1.4E+04	< 0.0001
	K	Africa	Americas	0.76	0.73	0.03	0.03	0.03	32.91	9.0E+03	3.2E+04	7E-229
	K	Africa	Asia	0.76	0.79	-0.03	-0.04	-0.03	-39.21	9.0E+03	1.5E+04	1.11e-317
	K	Americas	Asia	0.73	0.79	-0.07	-0.07	-0.06	-110.78	3.2E+04	1.5E+04	< 0.0001
	Mg	Africa	Americas	0.27	0.27	0.01	0.01	0.01	25.66	9.0E+03	3.2E+04	8E-142
	Mg	Africa	Asia	0.27	0.28	-0.01	-0.01	-0.01	-27.34	9.0E+03	1.4E+04	3E-161
	Mg	Americas	Asia	0.27	0.28	-0.01	-0.01	-0.01	-61.33	3.2E+04	1.4E+04	< 0.0001
	N	Africa	Americas	2.15	2.15	0.01	0.00	0.01	5.92	9.1E+03	3.2E+04	1E-08
	N	Africa	Asia	2.15	2.15	0.00	0.00	0.01	4.32	9.1E+03	1.4E+04	5E-05
	N	Americas	Asia	2.15	2.15	0.00	0.00	0.00	-1.35	3.2E+04	1.4E+04	0.534
	P	Africa	Americas	0.11	0.10	0.00	0.00	0.00	11.29	8.8E+03	3.2E+04	6E-29
	P	Africa	Asia	0.11	0.11	0.00	-0.01	0.00	-39.74	8.8E+03	1.4E+04	< 0.0001
	P	Americas	Asia	0.10	0.11	-0.01	-0.01	-0.01	-84.42	3.2E+04	1.4E+04	< 0.0001
	SLA	Africa	Americas	133.94	132.01	1.93	1.82	2.04	33.64	9.1E+03	3.2E+04	5E-239
	SLA	Africa	Asia	133.94	130.61	3.33	3.19	3.46	48.92	9.1E+03	1.4E+04	< 0.0001

SLA	Americas	Asia	132.01	130.61	1.40	1.29	1.50	25.75	3.2E+04	1.4E+04	6E-144
Thickness	Africa	Americas	0.86	0.82	0.04	0.03	0.04	29.21	9.0E+03	3.2E+04	3E-182
Thickness	Africa	Asia	0.86	0.80	0.06	0.06	0.06	43.43	9.0E+03	1.4E+04	< 0.0001
Thickness	Americas	Asia	0.82	0.80	0.02	0.02	0.03	22.10	3.2E+04	1.4E+04	7E-107
WC	Africa	Americas	53.62	54.05	-0.42	-0.45	-0.40	-37.86	9.0E+03	3.2E+04	1E-299
WC	Africa	Asia	53.62	54.80	-1.18	-1.21	-1.16	-98.64	9.0E+03	1.5E+04	< 0.0001
WC	Americas	Asia	54.05	54.80	-0.76	-0.78	-0.74	-84.23	3.2E+04	1.5E+04	< 0.0001
WD	Africa	Americas	0.60	0.60	0.00	0.00	0.00	6.77	9.0E+03	3.2E+04	4E-11
WD	Africa	Asia	0.60	0.58	0.03	0.03	0.03	102.97	9.0E+03	1.4E+04	< 0.0001
WD	Americas	Asia	0.60	0.58	0.03	0.03	0.03	141.41	3.2E+04	1.4E+04	< 0.0001

Table S3. Results of the t-test analysis, with Bonferroni correction, testing for significance of differences between the community level trait values between tropical broadleaf wet and dry forest as classified by the RESOLVE Ecoregions dataset (ecoregions.appspot.com). Area: leaf area, C: leaf carbon concentration, Ca: leaf calcium concentration, FM: leaf fresh mass, DM: leaf dry mass, K: leaf potassium concentration, Mg: leaf magnesium concentration, N: leaf nitrogen concentration, P: leaf phosphorus concentration, SLA: specific leaf area, Thickness: leaf thickness, WC: leaf water content, WD: wood density. The dataset specifies if the full (Full) number of pixels from the predictions were used or a random 10% or 1% of them to test the effect of sample size on the t-test results. Group: wet or dry forest group being compared, Mean value: average community weight trait value, value difference: difference between average community weighted mean trait value from group 1 and group 2. Conf-low: lower confidence interval, Conf-High: highest confidence interval, Statistic: t-test statistic value, n1= sample size for group 1, n2=sample size for group 2, P-val adj.: adjusted P-value.

Dataset	Trait	Group 1	Group 2	Mean value 1	Mean value 2	Value difference	Conf-low	Conf-High	Statistic	n1	n2	P-val adj.
**Full**	Area	Dry	Wet	-15.9926	99.5384	115.5310	-16.0278	-15.9574	-890.3044	717237	4828806	< 0.0001
	C	Dry	Wet	-0.8939	46.6359	47.5298	-0.8950	-0.8928	-1598.1569	717237	4828806	< 0.0001
	Ca	Dry	Wet	0.1892	0.9991	0.8099	0.1889	0.1895	1437.1710	717237	4828805	< 0.0001
	DM	Dry	Wet	-0.0270	0.9764	1.0034	-0.0273	-0.0267	-163.2954	717237	4828805	< 0.0001
	FM	Dry	Wet	0.0396	3.5309	3.4913	0.0384	0.0408	64.5406	717237	4828805	< 0.0001
	K	Dry	Wet	0.1094	0.8465	0.7371	0.1093	0.1096	1378.7608	717237	4828805	< 0.0001
	Mg	Dry	Wet	0.0235	0.2915	0.2679	0.0235	0.0236	1085.8950	717237	4828805	< 0.0001
	N	Dry	Wet	0.0694	2.2109	2.1415	0.0692	0.0696	821.8391	717237	4828805	< 0.0001
	P	Dry	Wet	0.0125	0.1175	0.1049	0.0125	0.0126	1486.2069	717237	4828805	< 0.0001
	SLA	Dry	Wet	3.4040	134.9167	131.5127	3.3924	3.4156	575.9264	717237	4828805	< 0.0001
	Thickness	Dry	Wet	0.0123	0.8296	0.8173	0.0120	0.0126	80.4628	717237	4828805	< 0.0001
	WD	Dry	Wet	-0.0067	0.5886	0.5954	-0.0068	-0.0067	-276.7966	717237	4828805	< 0.0001
	WC	Dry	Wet	0.0517	54.2217	54.1701	0.0492	0.0541	41.4167	717237	4828805	< 0.0001
**10%**	Area	Dry	Wet	-15.9380	99.6284	115.5663	-16.0495	-15.8264	-279.9214	71724	482881	< 0.0001
	C	Dry	Wet	-0.8924	46.6369	47.5292	-0.8958	-0.8889	-504.7866	71724	482881	< 0.0001
	Ca	Dry	Wet	0.1898	0.9996	0.8099	0.1890	0.1906	457.0041	71724	482880	< 0.0001
	DM	Dry	Wet	-0.0277	0.9755	1.0032	-0.0287	-0.0267	-52.9301	71724	482880	< 0.0001
	FM	Dry	Wet	0.0359	3.5277	3.4918	0.0321	0.0397	18.5182	71724	482880	2.05E-76
	K	Dry	Wet	0.1095	0.8465	0.7370	0.1090	0.1100	436.3606	71724	482880	< 0.0001

	Mg	Dry	Wet	0.0235	0.2915	0.2679	0.0234	0.0237	343.2956	71724	482880	< 0.0001
	N	Dry	Wet	0.0694	2.2110	2.1416	0.0688	0.0699	260.0841	71724	482880	< 0.0001
	P	Dry	Wet	0.0126	0.1175	0.1049	0.0125	0.0126	471.4666	71724	482880	< 0.0001
	SLA	Dry	Wet	3.3809	134.8945	131.5135	3.3443	3.4175	181.0268	71724	482880	< 0.0001
	Thickness	Dry	Wet	0.0129	0.8303	0.8173	0.0120	0.0139	26.7980	71724	482880	1.4E-157
	WD	Dry	Wet	-0.0068	0.5886	0.5954	-0.0069	-0.0066	-88.4168	71724	482880	< 0.0001
	WC	Dry	Wet	0.0503	54.2200	54.1697	0.0426	0.0581	12.7505	71724	482880	3.32E-37
	Area	Dry	Wet	-15.8223	99.6509	115.4732	-16.1778	-15.4667	-87.2251	7172	48288	< 0.0001
	C	Dry	Wet	-0.8957	46.6333	47.5290	-0.9067	-0.8848	-160.0554	7172	48288	< 0.0001
	Ca	Dry	Wet	0.1886	0.9981	0.8095	0.1860	0.1911	145.3307	7172	48288	< 0.0001
	DM	Dry	Wet	-0.0265	0.9770	1.0035	-0.0298	-0.0233	-16.0318	7172	48288	4.73E-57
	FM	Dry	Wet	0.0473	3.5345	3.4872	0.0351	0.0594	7.6195	7172	48288	2.81E-14
	K	Dry	Wet	0.1089	0.8460	0.7372	0.1073	0.1104	135.5608	7172	48288	< 0.0001
**1%**	Mg	Dry	Wet	0.0237	0.2917	0.2681	0.0233	0.0241	110.1227	7172	48288	< 0.0001
	N	Dry	Wet	0.0706	2.2119	2.1413	0.0690	0.0723	84.2816	7172	48288	< 0.0001
	P	Dry	Wet	0.0126	0.1175	0.1049	0.0124	0.0127	149.2620	7172	48288	< 0.0001
	SLA	Dry	Wet	3.4282	134.9368	131.5086	3.3121	3.5444	57.8361	7172	48288	< 0.0001
	Thickness	Dry	Wet	0.0109	0.8283	0.8174	0.0079	0.0139	7.0841	7172	48288	1.51E-12
	WD	Dry	Wet	-0.0065	0.5888	0.5953	-0.0070	-0.0060	-26.6720	7172	48288	5.7E-152
	WC	Dry	Wet	0.0728	54.2358	54.1630	0.0483	0.0973	5.8193	7172	48288	6.10E-09

Table S4. Principal component analysis of the functional trait space found across the tropical American, African and Asian forests. Values in each column for each principal component show the loadings of the traits for each given PC. PC: principal component, Area: leaf area, C: leaf carbon concentration, Ca: leaf calcium concentration, FM: leaf fresh mass, DM: leaf dry mass, K: leaf potassium concentration, Mg: leaf magnesium concentration, N: leaf nitrogen concentration, P: leaf phosphorus concentration, SLA: specific leaf area, Thickness: leaf thickness, WC: leaf water content, WD: wood density.

Trait	PC1	PC2	PC3	PC4	PC5	PC6	PC7	PC8	PC9	PC10	PC11	PC12	PC13
**Area**	0.10	0.47	0.38	0.05	0.22	0.16	0.04	0.09	0.22	0.17	0.35	0.58	0.07
**C**	0.35	0.13	-0.12	0.06	0.28	-0.49	0.09	0.39	0.42	-0.38	-0.11	-0.11	-0.10
**Ca**	-0.40	-0.06	-0.02	-0.08	-0.20	-0.14	-0.03	0.09	0.10	-0.15	0.64	-0.07	-0.57
**DM**	-0.03	0.53	0.19	-0.15	-0.29	-0.36	0.29	0.21	-0.54	0.08	-0.11	-0.13	-0.03
**FM**	-0.21	0.44	0.22	0.09	-0.13	0.42	0.07	-0.21	0.31	-0.41	-0.23	-0.37	-0.07
**K**	-0.39	0.01	-0.12	-0.01	-0.23	-0.18	-0.05	0.16	0.14	-0.24	0.23	0.04	0.77
**Mg**	-0.38	-0.07	-0.09	0.00	0.07	-0.18	0.63	-0.14	0.39	0.44	-0.20	0.03	-0.03
**N**	-0.26	-0.16	0.49	0.01	0.17	0.04	-0.32	0.53	0.10	0.33	-0.14	-0.33	0.02
**P**	-0.39	0.01	-0.12	0.02	-0.10	-0.01	-0.22	0.22	-0.05	-0.22	-0.52	0.59	-0.25
**SLA**	-0.17	-0.32	0.45	-0.15	0.46	-0.07	0.32	-0.14	-0.29	-0.45	0.02	0.09	0.06
**Thickness**	0.01	0.12	-0.10	-0.95	0.11	0.04	-0.16	-0.07	0.16	0.04	-0.06	-0.01	0.02
**WC**	-0.19	0.20	-0.51	0.04	0.47	0.41	0.19	0.37	-0.27	0.00	0.12	-0.13	0.02
**WD**	0.28	-0.31	0.09	-0.18	-0.44	0.41	0.43	0.46	0.08	-0.11	0.01	0.11	0.00
**Standard deviation**	2.39	1.63	1.32	1.01	0.7	0.58	0.47	0.45	0.41	0.38	0.31	0.27	0.2
**Proportion of Variance**	0.43	0.2	0.13	0.07	0.03	0.02	0.01	0.01	0.01	0.01	0.007	0.005	0.003
**Cumulative Proportion**	0.43	0.64	0.78	0.86	0.89	0.92	0.94	0.95	0.97	0.98	0.99	0.99	1

Table S5. An example of the EarthEngine code used to obtained the satellite Senitnel-2 raw spectral raw bands, calculate the vegetation indices, grey level co-occurrence matrix indices and extract the climatic, soil and terrain variables. The code has been simplified assuming all the assets needed are already in the workspace.

```
function maskS2clouds(image) {
  var qa = image.select('QA60');

  // Bits 10 and 11 are clouds and cirrus, respectively.
  var cloudBitMask = 1 << 10;
  var cirrusBitMask = 1 << 11;

  // Both flags should be set to zero, indicating clear conditions.
  var mask = qa.bitwiseAnd(cloudBitMask).eq(0)
      .and(qa.bitwiseAnd(cirrusBitMask).eq(0));

  return image.updateMask(mask).divide(10000);
}

function maskEdges(s2_img) {
  return s2_img.updateMask(
      s2_img.select('B8A').mask().updateMask(s2_img.select('B9').mask()));
}


var MAX_CLOUD_PROBABILITY = 25;
function maskClouds(img) {
  var clouds = ee.Image(img.get('cloud_mask')).select('probability');
  var isNotCloud = clouds.lt(MAX_CLOUD_PROBABILITY);
  return img.updateMask(isNotCloud);
}
```

```
///////////////////////////////////////////////////////////// Study area sample
var polygon = Gabon
////////////////////////////////////////////////////////////
////////////// OBTAIN THE SENTINEL-2 /////////////////////
////////////////////////////////////////////////////////////


// Map the function over the years of data and take the median.
// Load Sentinel-2 BOA reflectance data.
// ==============================================================================================================
// CHANGE THE MONTHS TO GET DATA FROM DEPENDING ON THE AREA OF INTEREST STATED ABOVE
var collection = ee.ImageCollection('COPERNICUS/S2_SR')
        .filterDate('2019-01-01', '2022-12-30')
        .filter(ee.Filter.calendarRange(5,11,'month'))
        .filter(ee.Filter.lt('CLOUDY_PIXEL_PERCENTAGE',25))
        .map(maskS2clouds)
        .map(maskEdges)
        .filterBounds(polygon);


var s2Clouds = ee.ImageCollection('COPERNICUS/S2_CLOUD_PROBABILITY')
.filterDate('2019-01-01', '2022-12-30')
.filter(ee.Filter.calendarRange(5,11,'month'))
.filterBounds(polygon);

// Join S2 SR with cloud probability dataset to add cloud mask.
var s2SrWithCloudMask = ee.Join.saveFirst('cloud_mask').apply({
```

```
  primary: collection,
  secondary: s2Clouds,
  condition:
      ee.Filter.equals({leftField: 'system:index', rightField: 'system:index'})
});


function clp(img) {
  return img.clip(polygon)
}

var Sen_AllBandsMedian =ee.ImageCollection(s2SrWithCloudMask).map(maskClouds).map(clp).median();
var Sen_AllBandsMedian = Sen_AllBandsMedian.select('B1', 'B2', 'B3', 'B4', 'B5', 'B6', 'B7', 'B8', 'B8A', 'B9', 'B11', 'B12');//selected bands
var visualization = {min: 0.0,max: 0.3,bands: ['B4', 'B3', 'B2'],};
Map.addLayer(Sen_AllBandsMedian,visualization,'Median1' )


///////////////////////////////////////////////////////
// VEGETATION INDICES                                /////
///////////////////////////////////////////////////////

// MCARI -This function gets Modified Chlorophyll Absorption in Reflectance Index -MCARI
var image_MCARI = Sen_AllBandsMedian.expression(
    '((B5 - B4) - 0.2 * (B5 - B3)) * (B5 / B4)', {
      'B4': Sen_AllBandsMedian.select('B4'),
      'B3': Sen_AllBandsMedian.select('B3'),
      'B5': Sen_AllBandsMedian.select('B5')
    });
var image_MCARI = image_MCARI.select(['B5'],['MCARI']);
```

```
//MSAVI 2 https://wiki.landscapetoolbox.org/doku.php/remote_sensing_methods:modified_soil-adjusted_vegetation_index
var image_MSAVI2 = Sen_AllBandsMedian.expression(
   '(2 * B8 + 1 - sqrt (pow((2 * B8 + 1),2) - 8 * (B8 - B4)))/2', {
     'B4': Sen_AllBandsMedian.select('B4'),
     'B8': Sen_AllBandsMedian.select('B8')
   });
var image_MSAVI2 = image_MSAVI2.select(['constant'],['MSAVI2']);

// NDRE -This function gets Normalized Difference Red edge Index
var image_NDRE = Sen_AllBandsMedian.expression(
   '((B8 - B5)/(B8 + B5))', {
     'B8': Sen_AllBandsMedian.select('B8'),
     'B5': Sen_AllBandsMedian.select('B5')
   });
var image_NDRE = image_NDRE.select(['B8'],['NDRE']);


/////////////////////////////////////////////////////////
// GRAY LEVEL CO-OCCURRENCE MATRIX of indices/////
/////////////////////////////////////////////////////////
// GLCM MCARI
var image_MCARI_32=image_MCARI.multiply(100).int32()
var glcm_MCARI = image_MCARI_32.glcmTexture({size: 9});
var corr_MCARI = glcm_MCARI.select('MCARI_corr');
var ent_MCARI = glcm_MCARI.select('MCARI_ent');

// GLCM MSAVI
var image_MSAVI_32=image_MSAVI2.multiply(100).int32()
var glcm_MSAVI = image_MSAVI_32.glcmTexture({size: 9});
var corr_MSAVI = glcm_MSAVI.select('MSAVI2_corr');
```

```
var ent_MSAVI = glcm_MSAVI.select('MSAVI2_ent');

// GLCM NDRE
var image_NDRE_32=image_NDRE.multiply(100).int32()
var glcm_NDRE = image_NDRE_32.glcmTexture({size: 9});
var corr_NDRE = glcm_NDRE.select('NDRE_corr');
var ent_NDRE = glcm_NDRE.select('NDRE_ent');


////////////////////////////////////////////////////////
// ADD BANDS TO CREATE ONE SINGLE RASTER STACK
////////////////////////////////////////////////////////

var  indices=image_MCARI.addBands(image_MSAVI2).addBands(image_NDRE).addBands(ent_NDRE).addBands(corr_MCARI).addBands(corr_MSAVI)
.addBands(corr_NDRE).addBands(ent_MCARI).addBands(ent_MSAVI).float();

/////////////////////////////////////////////////////////////
////// CLIMATIC DATA ---- TERRA CLIMATE
/////////////////////////////////////////////////////////////
////////////////////////////////////////////////////////////

///////////////////////
//SRAD
var months = ee.List.sequence(1,12);
var dataset = ee.ImageCollection('IDAHO_EPSCOR/TERRACLIMATE')
               .filter(ee.Filter.date('1989-01-01', '2018-12-31'));
var byMonth_srad = ee.ImageCollection.fromImages(
    months.map(function(m) {
      return dataset.filter(ee.Filter.calendarRange(m, m, 'month'))
                 .select('srad').mean()
```

```
                  .set('month', m);
}));
var byYear_srad_mean = byMonth_srad.reduce(ee.Reducer.mean()).multiply(.1).mask(cwd_1k).select(
    ['srad_mean'],['b1_2']);

/////////////////////
//MCWD
var months = ee.List.sequence(1,12);
var dataset = ee.ImageCollection('IDAHO_EPSCOR/TERRACLIMATE')
                 .filter(ee.Filter.date('1989-01-01', '2017-12-31'));

var byMonth_cwd = ee.ImageCollection.fromImages(
      months.map(function(m) {
        return dataset.filter(ee.Filter.calendarRange(m, m, 'month'))
                  .select('def').max()
                  .set('month', m);
}));

var byYear_cwd_mean = byMonth_cwd.reduce(ee.Reducer.mean()).multiply(.1)
.mask(cwd_1k).select(
    ['def_mean'],['b1']);

/////////////////////

var byYear_def_mean =   cwd_1k
var byYear_tmax_mean=   tmax_1k
var byYear_srad_mean=   byYear_srad_mean
var byYear_smoist_mean= smoist_1k
var climate = byYear_def_mean.addBands(byYear_tmax_mean).addBands(byYear_srad_mean).addBands(byYear_smoist_mean);
var climate = climate.select(['b1','b1_1','b1_2','b1_3'], ['def_mean','tmmx_mean','srad_mean','soil_mean']);
```

```
////////////////////////////////////////////////////////////////////////////////////////////////////////////////////////////////////////////
////// SOIL and SLOPE
////////////////////////////////////////////////////////////////////////////////////////////////////////////////////////////////////////////
////////////////////////////////////////////////////////////////////////////////////////////////////////////////////////////////////////////

// Calculate slope
var Slope = ee.Terrain.slope(elevation)

//FROM SOIL GRIDS directly
var ph_sg1 = ee.Image("projects/soilgrids-isric/phh2o_mean").select('phh2o_0-5cm_mean','phh2o_5-15cm_mean','phh2o_15-30cm_mean')
.reduce(ee.Reducer.mean())
var cec_sg1 = ee.Image("projects/soilgrids-isric/cec_mean").select('cec_0-5cm_mean','cec_5-15cm_mean','cec_15-30cm_mean')
.reduce(ee.Reducer.mean()).divide(10);
var clay_sg1 = ee.Image("projects/soilgrids-isric/clay_mean").select('clay_0-5cm_mean','clay_5-15cm_mean','clay_15-30cm_mean')
.reduce(ee.Reducer.mean()).divide(10);
var sand_sg1 = ee.Image("projects/soilgrids-isric/sand_mean").select('sand_0-5cm_mean','sand_5-15cm_mean','sand_15-30cm_mean')
.reduce(ee.Reducer.mean()).divide(10);
var soil = ph_sg1.addBands(sand_sg1).addBands(clay_sg1).addBands(cec_sg1)
var soil = soil.select(['mean', 'mean_1','mean_2','mean_3'], ['ph','sand','clay','cec']);


//////////////////////////////////////////////////////////////
//Add climate, slope, soil layers to Sentinel-2 stack /////
//////////////////////////////////////////////////////////////
var abiotic = climate.addBands(Slope).addBands(soil).float()


/////////////////////////////////////////////////////////////////////////
// CLIP AND MASKS LAYERS TO POLYGON
```

```
//////////////////////////////////////////////////////////////////////////////////
var Sen_AllBandsMedian = Sen_AllBandsMedian.addBands(indices).addBands(abiotic)
print(Sen_AllBandsMedian)

/////////////////////////////////////////////////////////////////////////////////////////////////////////////////////
// CLIP AND MASKS LAYERS TO POLYGON, ECOREGION AND HANSEN tree cover///////////////
// Create vegetation mask and limit the products to it ///////////////////////////////////////////////
///////////////////////////////////////////////////////////////////////////////////////////////////////////////////
var ecoregions1 = ecoregions.filterMetadata('BIOME_NUM','equals', 1)//Tropical & Subtropical Moist Broadleaf Forests
var ecoregions2 = ecoregions.filterMetadata('BIOME_NUM','equals', 2)//Tropical & Subtropical Dry Broadleaf Forests
var ecoregions4 = ecoregions1.merge(ecoregions2);
var ecoregions4 = ecoregions4.filterBounds(polygon)

var treeCover = hansen.select(['treecover2000']).gte(25).clip(polygon).clip(ecoregions4)//At least 25% tree cover in the pixel

// Create vegetation mask and limit the products to it
var ESA2021_1 = ESA2021.filterMetadata('MAP','equals', 10)
var ESA2021_2 = ESA2021.filterMetadata('MAP','equals', 20)
var ESA2021_3 = ESA2021_1.merge(ESA2021_2).filterBounds(polygon).filterBounds(ecoregions4);

///////////////////////////////////////////////////////////////////////////////////////////////////////////////////
/////////////////////////    Clip the original layers ////////////////////////////////////////////////////////////
/////////////////////////////////////////////////////////////////////////////////////////////////////////////////////
var Sen_AllBandsMedian = Sen_AllBandsMedian.clip(polygon).clip(ecoregions4)//.clip(ESA2021_3)

Map.addLayer(Sen_AllBandsMedian.select('B8A'))
Map.addLayer(Sen_AllBandsMedian)

Export.image.toDrive({
   image: Sen_AllBandsMedian,
```

```
    description: 'Gabon_10m',
    folder: 'S2_FP_2023',
    fileNamePrefix: 'NONE',
    //region: polygon.geometry().bounds(),
    scale: 10,
    maxPixels:1e13,
    skipEmptyTiles:true,
    fileFormat:'GeoTIFF'
  })
//End of code
```
